# Novel Triarylamine-Based Hole Transport Materials: Synthesis, Characterization and Computational Investigation

**DOI:** 10.3390/ma14113128

**Published:** 2021-06-07

**Authors:** Laila M. Nhari, Reda M. El-Shishtawy, Qiuchen Lu, Yuanzuo Li, Abdullah M. Asiri

**Affiliations:** 1 Chemistry Department, Faculty of Science, King Abdulaziz University, Jeddah 21589, Saudi Arabia; lnahary0001@stu.kau.edu.sa; 2 Chemistry Department, Faculty of Science, University of Jeddah, Jeddah 21589, Saudi Arabia; 3 Dyeing, Printing and Textile Auxiliaries Department, Textile Research Division, National Research Centre, Dokki, Cairo 12622, Egypt; 4 College of Science, Northeast Forestry University, Harbin 150040, China; qiuchenlu1997@126.com; 5 Center of Excellence for Advanced Materials Research, King Abdulaziz University, Jeddah 21589, Saudi Arabia; aasiri2@kau.edu.sa

**Keywords:** 2-acetylphenothiazine, hole transport material, perovskite solar cell, triarylamine, hole mobility, density functional theory

## Abstract

Three novel triarylamine-based electron-rich chromophores were synthesized and fully characterized. **Compounds 1** and **2** were designed with electron-rich triphenylamine skeleton bearing two and four decyloxy groups namely, 3,4-bis(decyloxy)-*N,N*-diphenylaniline and *N*-(3,4-bis(decyloxy)phenyl)-3,4-bis(decyloxy)-*N*-phenylaniline, respectively. The well-known electron-rich phenothiazine was introduced to diphenylamine moiety through a thiazole ring to form *N,N*-bis(3,4-bis(decyloxy)phenyl)-5-(10*H*-phenothiazin-2-yl)thiazol-2-amine (**Compound 3**). These three novel compounds were fully characterized and their UV–vis absorption indicated their transparency as a favorable property for hole transport materials (HTMs) suitable for perovskite solar cells. Cyclic voltammetry measurements revealed that the HOMO energy levels were in the range 5.00–5.16 eV for all compounds, indicating their suitability with the HOMO energy level of the perovskite photosensitizer. Density functional theory (DFT) and time-dependent DFT (TD-DFT) have been used to investigate the possibility of the synthesized compounds to be utilized as HTMs for perovskite solar cells (PSCs). The computational investigation revealed that the hole mobility of **Compound 1** was 1.08 × 10^−2^ cm^2^ V^−1^ s^−1^, and the substitution with two additional dialkoxy groups on the second phenyl ring as represented by **Compound 2** significantly boosted the hole mobility to reach the value 4.21 × 10^−2^ cm^2^ V^−1^ s^−1^. On the other hand, **Compound 3**, in which the third phenyl group was replaced by a thiazole-based phenothiazine, the value of hole mobility decreased to reach 5.93 × 10^−5^ cm^2^ V^−1^ s^−1^. The overall results indicate that these three novel compounds could be promising HTMs for perovskite solar cells.

## 1. Introduction

Hole transport materials (HTMs) represent the type of organic electron-rich compounds with a sufficient length of an extended π-conjugated system having reasonable planarity to enhance its role in transporting holes from the adsorbent to cathode in organic electronic devices [1]. The ideal skeleton of an HTM consists of a central aromatic core linked to one or more than one electron-rich terminal moieties. Effective HTMs must have several properties such as low cost, good stability, infinite variety, environmental friendless, solution-processability, mechanical flexibility, tenability of electronic characteristics and easy fabrication [2,3]. HTMs represent fundamental building blocks for perovskite solar cells owing to its role in accelerating and increasing the hole extraction from the perovskite material (methylammonium lead halides (CH_3_NH_3_PbX_3_, where X=Cl, Br, or I) to conduct holes to the cathode [4,5,6]. To generate a sufficient driving force for hole extraction in PSCs, the highest occupied molecular orbital (HOMO) level of efficient HTMs must be higher than the valence band (VB) of perovskite (i.e., the absolute value of HOMO level of HTMs must be lower than the absolute value of the VB of perovskite).

Tuning energy levels of HTMs, especially the HOMO level, can be achieved by modifying HTMs molecular structures to maximize the photovoltaic performance of PSCs [7]. Numerous studies have been done to fulfill this issue using different techniques such as: structural tuning [8,9,10,11], changing numbers and positions of attached methoxy groups [12,13], conjugation with more electron-donating moieties [14,15,16], replacing the core of spiro-OMeTAD with other electron-rich cores [17,18], attaching aromatic groups onto their peripheral positions [19,20], binary HTMs blending [21], etc. Fabrication of the first highly efficient PSC was accomplished using the common spiro-OMeTAD as an HTM producing high power conversion efficiency (PCE) > 20%. Nevertheless, the high cost, multistep synthesis process and poor device stability related to spiro-OMeTAD hindered its practical application and required more efforts to design new HTMs [7]. On the other hand, computational chemistry is capable of providing profitable insights to predict the molecular electronic structures and optical properties of newly designed molecules and its suitability as HTMs for PSCs [22,23,24,25].

The triarylamine core has proven great potential as a candidate for constructing optoelectronics owing to its three-dimensional structure that would hamper molecular aggregation and its beneficial high electron density that can be tuned by structural modification according to the need of the application [26,27]. Therefore, it was envisioned to design and synthesize different triarylamine derivatives containing two and four *ortho*-alkoxy long chains, with phenyl or phenothiazinyl moieties. The newly synthesized triarylamine derivatives were fully characterized and their optoelectronic computation as HTMs were also investigated. Herein, we reported the synthesis, characterization, optoelectronic properties and computational investigation of novel triarylamine-based HTMs for PSCs.

## 2. Experimental

### 2.1. General

All solvents were purchased from Sigma-Aldrich and Fisher and used directly without further purification. All other chemicals used herein were of analytical grade and were used without further purification. Chromatographic separations were carried out on silica gel (60–120 mesh). ^1^H and ^13^C NMR spectra were recorded in CDCl_3_ or DMSO-*d*_6_ on a Bruker Avance 400 and 850 MHz and 100 and 213 MHz spectrometer respectively. The reported chemical shifts were against TMS. Infrared spectra (FT-IR) were performed on a Perkin-Elmer spectrum 100 FTIR spectrometer. Mass spectra were recorded on a positive ion mode on a Bruker Impact II, LC–MS/MS. Melting point apparatus SMP3 was uncorrected. UV–visible absorption spectra were recorded with a Jasco V560 spectrophotometer (Jasco International Co., Ltd., Tokyo, Japan). Cyclic voltammetric studies of the compounds were carried out in dichloromethane solution (about 10^−3^ M) containing 0.1 M tetrabutylammonium hexafluorophosphate (n-Bu_4_NPF_6_) as the supporting electrolyte at room temperature on an Autolab PGSTAT204 potentiostat system using a three-electrode cell under nitrogen atmosphere. The conventional three electrodes were the platinum sheet as a counter electrode, glassy carbon as the working electrode and Ag/AgCl as a reference electrode. The reference electrode potential was calibrated against ferrocene/ferrocenium (Fc/Fc+) after each voltammetry run. The solution was anodically and catholically scanned with a rate of 50 mV s^−1^.

### 2.2. Computational Methods

All calculations were performed by the Gaussian 09 program [28]. Density functional theory (DFT) [29] with B3LYP [30] functional at the 6–31G(d) basis set was used to optimize the ground-state geometries of **Compounds 1–3** (see Figure S1 in Supplementary Materials) and ground-state structure and energy levels were obtained under the same framework. The conductor polarizable continuum model (CPCM) [31] was used to demonstrate the solvent effect of dichloromethane. Time-dependent DFT (TD-DFT) [32] with the CAM-B3LYP [33] and the 6–31G(d) basis set were used to acquire the corresponding absorption and fluorescence spectrum. Additionally, the optimization of excited states was done under DFT/CAM-B3LYP/6–31G(d) level. Reorganization energy was calculated using optimized charged molecules with DFT/B3LYP/6–31G(d). The face-to-face model in the HTM dimer was used to calculate the charge transfer integral. The density of states (DOS) and partial density of states (PDOS) were analyzed using GaussSum software [28,34]. Furthermore, Marcus theory was employed to study the hole mobility of the three compounds.

The absolute hardness (*η*) was used to estimate the stability of cells using the following equation [35]:

(1) $$\eta =\frac{IP-EA}{2}$$

The fluorescence lifetime *τ*_1_ can be calculated by the speed of light *c*, oscillator strength *f* and fluorescence energy *E* [36]:

(2) $$\tau_{1}=\frac{ac^{3}u^{2}}{2fE^{2}}$$

The excited-state lifetime *τ*_2_ can be calculated by the oscillator strength *f* and the vertical excitation energy *E* [37]:

(3) $$\tau_{2}=\frac{1.499}{fE^{2}}$$

The reorganization energy (*λ_h/e_*) refers to the energy change of the system, which is caused by the structural relaxation after the gain or loss of electrons [38,39]. It can be calculated by:

(4) $$\lambda_{h/e}=(E_{0}^{\pm }-E_{\pm })+(E_{\pm }^{0}-E_{0})$$

where$\;E_{0}^{+}\left(E_{0}^{-}\right)$ is the energy of the neutral molecule under the cation (anion) and $E_{+}^{0}\;\left(E_{-}^{0}\right)$ is the energy of cationic (anionic) molecule under the neutral state, $E_{+}\;\left(E_{-}\right)$ represents the optimized energy under the cation (anion) and $E_{0}$ is the ground-state energy of the neutral molecule.

When the dimer configuration is completely parallel to the face-to-face transport configuration, the charge transfer integral (*V_h/e_*) can be considered as the half of difference of energy between HOMO (LUMO+1) and HOMO−1 (LUMO) of two adjacent neutral dimers. At this time, the error of the calculated charge transfer integral is 0%. So, the charge transfer integral (*V_h/e_*) can be calculated using the following equations [40]:

(5) $$V_{h}=\frac{E_{HOMO}-E_{HOMO-1}}{2}$$

(6) $$V_{e}=\frac{E_{LUMO+1}-E_{LUMO}}{2}$$

where *E* means the corresponding orbital energy. Marcus theory was used to calculate the charge hopping rate [41]:

(7) $$k_{h/e}=\frac{v_{h/e}^{2}}{\hbar }\sqrt{\frac{\pi }{\lambda_{h/e}k_{B}T}}\exp(-\frac{\lambda_{h/e}}{4k_{B}T})$$

where *k_B_*, *v_h/e_*, *T* and *λ_h/e_* are represented as the Boltzmann constant, hole/electron transfer integral, room temperature (300 K) and hole/electron reorganization energy, respectively.

Mobility can be approximately calculated by the Einstein relation [42]:

(8) $$\mu =\frac{e}{k_{B}T}\frac{1}{2n}\sum_{i}r_{i}^{2}k_{i}P_{i}$$

where *n* is the dimensionality (*n* = 1) and *r*_i_ is centroid to centroid distance.

### 2.3. Synthesis

#### 2.3.1. 1,2-Bis(decyloxy)benzene (**B1**)

To DMSO (2 mL) was added powdered KOH (2.2 g, 40 mmol). After stirring for 5 min, catechol (1.1 g, 10 mmol) was added, followed immediately by the decyl bromide (4.4 g, 20 mmol). Stirring was continued for 30 min after which the mixture was poured into water (200 mL) and extracted with dichloromethane (3 × 200 mL). The combined organic extracts were then dried over anhydrous sodium sulfate, filtered and the filtrate was vacuum evaporated. Recrystallization of the residue from methanol gave the product **B1** in good yield (2.4 g, 61%) white crystals (mp. 39 °C). ^1^H NMR; (850 M Hz, CDCl_3_) δ: 6.87–6.89 (*m*, 4H, Ar-H), 3.99 (*t*, 4H, 2OCH_2_, *J* = 6.8 Hz), 1.81 (*quint*, 4H, 2CH_2_, *J* = 6.8 Hz), 1.46 (*quint*, 4H, 2CH_2_, *J* = 6.8 Hz), 1.36 (*quint*, 4H, 2CH_2_, *J* = 6.8 Hz), 1.24–1.31 (*m*, 20H, 10CH_2_), 0.88 (*t*, 6H, 2CH_3_, *J* = 6.8 Hz). ^13^C NMR; (213 M Hz, CDCl_3_) δ: 149.24, 120.99, 114.11, 69.28, 31.92, 29.65, 29.60, 29.45, 29.36, 29.35, 26.06, 22.69, 14.12. IR; υ cm^−1^: C-H aliphatic 2916, 2848, C=C stretch 1594. MS (ESI): *m/z* calcd for *C*_26_*H*_47_*O*_2_ 391.4 [M+H]^+^, found 391.2.

#### 2.3.2. 1,2-Bis(decyloxy)-4-iodobenzene (**B2**)

Compound (**B1**) (3.9 g, 10 mmol) was dissolved in boiling glacial acetic acid (50 mL), and potassium iodide (1.16 g, 7 mmol) and potassium iodate (0.85 g, 4 mmol) were added, and the mixture then boiled until iodine color disappear. The hot solution was decanted from the undissolved potassium iodate and allowed to cool slowly to room temperature. After filtration, 5% of sodium hydrogen sulphite (300 mL) was used for washing to obtain a white solid product. Then, the product was washed with plenty of water. Recrystallization from methanol afforded the product **B2** in an excellent yield (4.8 g, 93%) white crystals (mp. 50 °C). ^1^H NMR; (850 M Hz, CDCl_3_) δ: 7.17 (*dd*, 1H, Ar-H, *J* = 8.5, 2.55 Hz), 7.12 (*d*, 1H, Ar-H, *J* = 1.7 Hz), 6.61 (*d*, 1H, Ar-H, *J* = 8.5 Hz), 3.95 (*td*, 4H, 2OCH_2_, *J* = 6.8, 1.7 Hz), 1.79 (*sixtet*, 4H, 2CH_2_, *J* = 6.8 Hz), 1.45 (*sixtet*, 4H, 2CH_2_, *J* = 7.65 Hz), 1.41 (*sixtet*, 4H, 2CH_2_, *J* = 7.65 Hz), 1.26–1.31 (*m*, 20H, 10CH_2_), 0.88 (*overlapped-t*, 6H, 2CH_3_, *J* = 6.8, 1.7 Hz). ^13^C NMR; (213 M Hz, CDCl_3_) δ: 150.12, 149.23, 129.79, 122.64, 115.71, 82.50, 69.42, 69.35, 31.91, 29.61, 29.57, 29.39, 29.37, 29.34, 29.17, 29.16, 25.97, 22.69). IR; υ cm^−1^: C-H aliphatic 2919, 2848, C = C stretch 1584. MS (ESI): *m/z* calcd for *C*_26_*H*_46_*IO*_2_ 517.3 [M+H]^+^, found 517.3.

#### 2.3.3. 3,4-Bis(decyloxy)-N,N-diphenylaniline (**Compound 1**)

Into a three-neck round-bottomed flask was added a mixture of diphenylamine (2.5 g, 15 mmol), compound (**B2**) (5.2 g, 10 mmol), 1,10-phenanthroline (5.4 g, 30 mmol) and CuI (5.7 g, 30 mmol) in 30 mL of *p*-xylene. The temperature was increased to 100 °C, followed by the addition of potassium hydroxide (3.4 g, 60 mmol). The mixture was refluxed with stirring for 6 h. Then, the mixture was cooled down to room temperature, and then 50 mL of toluene was poured into the mixture. The mixture was filtered after stirring, evaporated under vacuum and purified by column chromatography using petroleum ether as an eluent to give **Compound 1** (2.9 g, 52%) white powder (mp. 48 °C). ^1^H NMR; (850 M Hz, CDCl_3_) δ: 7.19–7.22 (*m*, 4H, Ar-H), 7.04 (*dd*, 4H, Ar-H, *J* = 8.5, 0.85 Hz), 6.93–6.95 (*m*, 2H, Ar-H), 6.78 (*d*, 1H, Ar-H, *J* = 8.5 Hz), 6.69 (*d*, 1H, Ar-H, *J* = 2.55 Hz), 6.61 (*dd*, 1H, Ar-H, *J* = 8.5, 2.55 Hz), 3.98 (*t*, 2H, OCH_2_, *J* = 6.8 Hz), 3.84 (*t*, 2H, OCH_2_, *J* = 6.8 Hz), 1.80 (*quint*, 2H, CH_2_, *J* = 6.8 Hz), 1.73 (*quint*, 2H, CH_2_, *J* = 6.8 Hz), 1.46 (*quint*, 2H, CH_2_, *J* = 7.65 Hz), 1.39 (*quint*, 2H, CH_2_, *J* = 7.65 Hz), 1.35 (*quint*, 2H, CH_2_, *J* = 7.65 Hz), 1.26–1.31 (*m*, 22H, 11CH_2_), 0.88 (*overlapped-t*, 6H, 2CH_3_, *J* = 7.65, 2.55 Hz). ^13^C NMR; (213 M Hz, CDCl_3_) δ: 149.79, 148.07, 145.89, 141.07, 129.01, 122.93, 121.81, 118.33, 114.66, 112.36, 69.65, 69.17, 31.92, 29.65, 29.60, 29.57, 29.47, 29.44, 29.36, 29.20, 26.09, 26.06, 25.99, 22.70, 14.13. IR; υ cm^−1^: C-H aliphatic 2919, 2849, C=C stretch 1583. HRMS (ESI): *m/z* calcd for *C*_38_*H*_56_*NO*_2_ 558.4311 [M+H]^+^, found 558.4306.

#### 2.3.4. N-(3,4-Bis(decyloxy)phenyl)-3,4-bis(decyloxy)-N-phenylaniline (**Compound 2**)

A suspension of (2.3 g, 25 mmol) of aniline (38.7 g, 75 mmol) of compound (**B2**) and 200 mL 1, 2-dichlorobenzene were added into a three-necked flask equipped with a magnetic stirrer, a reflux condenser, and a nitrogen input tube, and then (17.9 g, 130 mmol) of anhydrous potassium carbonate and (8.3 g, 130 mmol) copper powder was added slowly. At last, a catalytic amount of 18-crown-6 was added under stirred. The reaction mixture was refluxed for 24 h and monitored by TLC. After completion of the reaction, the solvent 1,2-dichlorobenzene was evaporated under reduced pressure after the solution was cooled to room temperature. The crude product was purified by column chromatography with petroleum ether/dichloromethane (9:1) to yield **Compound 2** (21.5g, 60%) of white powder (mp. 76 °C). ^1^H NMR; (850 M Hz, CDCl_3_) δ: 7.16–7.18 (*m*, 2H, Ar-H), 6.97 (*dd*, 2H, Ar-H, *J* = 8.5, 1.7 Hz), 6.87 (*t*, 1H, Ar-H, *J* = 7.65 Hz), 6.76 (*d*, 2H, Ar-H, *J* = 8.5 Hz), 6.67 (*d*, 2H, Ar-H, *J* = 2.55 Hz), 6.58 (*dd*, 2H, Ar-H, *J* = 8.5, 2.55 Hz), 3.96 (*t*, 4H, 2OCH_2_, *J* = 6.8 Hz), 3.83 (*t*, 4H, 2OCH_2_, *J* = 6.8 Hz), 1.79 (*quint*, 4H, 2CH_2_, *J* = 6.8 Hz), 1.73 (*quint*, 4H, 2CH_2_, *J* = 6.8 Hz), 1.46 (*quint*, 4H, 2CH_2_, *J* = 6.8 Hz), 1.40 (*quint*, 4H, 2CH_2_, *J* = 6.8 Hz), 1.25–1.37 (*m*, 48H, 24CH_2_), 0.88 (*overlapped-t*, 12H, 4CH_3_, *J* = 7.65 Hz). ^13^C NMR; (213 M Hz, CDCl_3_) δ: 149.68, 148.55, 145.30, 141.48, 128.83, 121.36, 120.66, 117.28, 114.71, 111.48, 69.73, 69.12, 31.92, 29.70, 29.65, 29.62, 29.60, 29.58, 29.47, 29.45, 29.36, 29.23, 26.09, 26.01, 22.69, 14.12. IR; υ cm^−1^: C-H aliphatic 2917, 2849, C=C stretch 1593. HRMS (ESI): *m/z* calcd for *C*_58_*H*_96_*NO*_4_ 870.7333 [M+H]^+^, found 870.7339.

#### 2.3.5. 1-(10-Dodecyl-10*H*-phenothiazin-2-yl)ethan-1-one (**A1**)

A mixture of 2-acetylphenothiazine (1.4 g, 6 mmol), alkyl iodide (3 g, 18 mmol), potassium hydroxide 40% (20 ml) and tetrabutylammonium iodide (TBAI) (0.66 g, 1.8 mmol) in 20 ml toluene was refluxed gently at 65 °C for 48 h with good stirring. After the reaction mixture was cooled to room temperature, 50 ml of water was added to the mixture by extraction with ethyl acetate (3 × 50 ml). The organic layer was washed with a saturated aqueous solution of ammonium chloride and then water. After drying the organic layer with sodium sulfate anhydrous and evaporation under reduced pressure, brown oil of the product was obtained. The product was purified by column chromatography (eluent; petroleum ether: ethyl acetate 98:2) on silica gel to give (**A1**) (1.6 g, 67%) as a yellow oil. ^1^H NMR; (850 M Hz, cdcl_3_) δ: 7.45 (*d*, 1H, Ar-H, *J* = 7.65 Hz), 7.42 (*s*, 1H, Ar-H), 7.15–7.17 (*m*, 2H, Ar-H), 7.09 (*d*, 1H, Ar-H, *J* = 7.65 Hz), 6.92 (*t*, 1H, Ar-H, *J* = 7.65 Hz), 6.86 (*d*, 1H, Ar-H, *J* = 8.5 Hz), 3.87 (*t*, 2H, NCH_2_, *J* = 6.8 Hz), 2.56 (*s*, 3H, CH_3_), 1.79 (*quint*, 2H, CH_2_, *J* = 7.65 Hz), 1.43 (*quint*, 2H, CH_2_, *J* = 7.65 Hz), 1.23–1.32 (*m*, 16H, 8CH_2_), 0.88 (*t*, 3H, CH_3_, *J* = 6.8 Hz). ^13^C NMR; (213 M Hz, cdcl_3_) δ: 197.47, 145.56, 144.56, 136.22, 132.03, 127.60, 127.40, 126.95, 123.59, 122.93, 122.68, 115.71, 113.89, 47.51, 31.91, 29.62, 29.53, 29.51, 39.34, 29.22, 26.90, 26.68, 26.59, 22.69, 14.13. IR; υ cm^−1^: C-H aliphatic 2922, 2852, C=O 1681, C=C stretch 1591, 1558. MS (ESI): *m/z* calcd for *C*_26_*H*_36_*NOS* 410.3 [M+H]^+^, found 410.2.

#### 2.3.6. 2-Bromo-1-(10-dodecyl-10*H*-phenothiazin-2-yl)ethan-1-one (**A2**)

Copper (II) bromide (2.5 g, 11 mmol) was placed in a round-bottom flask fitted with a reflux condenser, and ethyl acetate (20 mL) was added and brought to reflux. Compound (**A1**) (2.4 g, 10 mmol) was dissolved in hot chloroform (20 mL) and added to the flask. The resulting reaction mixture was gently refluxed at 60 °C with vigorous stirring until the disappearance of all black solids (30 min). After cooling and removal of the copper(1)bromide by filtration, the solvents were removed from the filtrate under reduced pressure. The residue was dissolved in 50 mL of chloroform then washed 3 times with sodium hydroxide solution 1% and then water. After drying the organic layer with sodium sulfate anhydrous and evaporation under reduced pressure, an orange oil was obtained. The product was purified by column chromatography (eluent; petroleum ether:dichloromethane; 7:3) on silica gel to give (**A2**) (4.8g, 98%) as an orange oil. ^1^H NMR; (850 M Hz, CDCl_3_) δ: 7.44 (*dd*, 1H, Ar-H, *J* = 7.65, 1.7 Hz), 7.42 (*d*, 1H, Ar-H, *J* = 1.7 Hz), 7.16–7.18 (*m*, 2H, Ar-H), 7.09 (*dd*, 1H, Ar-H, *J* = 7.65, 1.7 Hz), 6.93 (*td*, 1H, Ar-H, *J* = 7.65, 0.85 Hz), 6.86 (*d*, 1H, Ar-H, *J* = 7.65 Hz), 4.39 (*s*, 2H, CH_2_Br), 3.87 (*t*, 2H, NCH_2_, *J* = 7.65 Hz), 1.79 (*quint*, 2H, CH_2_, *J* = 7.65 Hz), 1.43 (*quint*, 2H, CH_2_, *J* = 7.65 Hz), 1.24–1.33 (*m*, 16H, 8CH_2_), 0.88 (*t*, 3H, CH_3_, *J* = 6.8 Hz). ^13^C NMR; (213 M Hz, CDCl_3_) δ: 190.76, 145.71, 144.31, 133.38, 132.91, 127.74, 127.41, 127.06, 123.23, 123.15, 122.85, 115.74, 114.58, 47.62, 31.92, 30.64, 29.71, 29.63, 29.54, 29.52, 29.35, 29.22, 26.90, 26.61, 22.70, 14.14. IR; υ cm^−1^: C-H aliphatic 2922, 2852, C=O 1676, C=C stretch 1591, 1558. MS (ESI): *m/z* calcd for *C*_26_*H*_35_*BrNOS* 488.2 [M+H]^+^, found 488.1.

#### 2.3.7. 5-(10-Dodecyl-10*H*-phenothiazin-2-yl)thiazol-2-amine (**A3**)

A mixture of thiourea (0.5 g, 7 mmol) and compound (**A2**) (3.4 g, 7 mmol) in 70 mL acetone was stirred overnight at room temperature and filtered to afford the corresponding hydrobromide salt quantitatively. Then, the salt was treated with 5% ammonia solution with stirring and filtered to give the desired thiazole product **A3** (3.16g, 99%) as a pale-yellow powder (mp. 80 °C). ^1^H NMR; (850 M Hz, CDCl_3_) δ: 7.32 (*d*, 1H, Ar-H, *J* = 1.7 Hz), 7.27 (*dd*, 1H, Ar-H, *J* = 8.5, 1.7 Hz), 7.12–7.15 (*m*, 2H, Ar-H), 7.09 (*d*, 1H, Ar-H, *J* = 8.5 Hz), 6.89 (*td*, 1H, Ar-H, *J* = 6.8, 0.85 Hz), 6.85 (*dd*, 1H, Ar-H, *J* = 8.5, 0.85 Hz), 6.66 (*s*, 1H, Ar-H), 5.09 (*s*, 2H, NH_2_), 3.89 (*t*, 2H, NCH_2_, *J* = 6.8 Hz), 1.81 (*quint*, 2H, *J* = 7.65 Hz), 1.43 (*quint*, 2H, CH_2_, *J* = 6.8 Hz), 1.22–1.32 (*m*, 16H, 8CH_2_), 0.87 (*t*, 3H, CH_3_, *J* = 6.8 Hz). ^13^C NMR; (213 M Hz, CDCl_3_) δ: 167.08, 151.05, 145.76, 145.13, 133.93, 127.37, 127.35, 127.16, 124.90, 124.50, 122.29, 119.91, 115.58, 113.33, 102.65, 47.44, 31.91, 29.64, 29.63, 29.56, 29.53, 29.34, 29.26, 26.96, 26.94, 22.69, 14.13. IR; υ cm^−1^: N-H 3281, 3126, C-H aliphatic 2914, 2849, C=N stretch 1705, C=C stretch 1532. MS (ESI): *m/z* calcd for *C*_27_*H*_36_*N*_3_*S*_2_ 466.2 [M+H]^+^, found 466.2.

#### 2.3.8. N,N-Bis(3,4-bis(decyloxy)phenyl)-5-(10-dodecyl-10*H*-phenothiazin-2-yl)thiazol-2-amine (**Compound 3**)

Into a three-neck round-bottomed flask was added a mixture of compound (**A3**) (2.3 g, 5 mmol), compound (**B2**) (5.2 g, 10 mmol), 1,10-phenanthroline (5.4 g, 30 mmol) and CuI (5.7 g, 30 mmol) in 30 mL of *p*-xylene. The temperature was increased to 100 °C, followed by the addition of potassium hydroxide (3.4 g, 60 mmol). The mixture was refluxed with stirring for 6 h. Then, the mixture was cooled down to room temperature, and then 50 mL of toluene was poured into the mixture. The mixture was filtered after stirring, evaporated under vacuum and purified by column chromatography using petroleum ether and dichloromethane 8:2 as an eluent to afford **Compound 3** (3 g, 49%) as a yellow semisolid. ^1^H NMR; (850 M Hz, CDCl_3_) δ: 7.55 (*d*, 2H, Ar-H, *J* = 1.7 Hz), 7.50 (*dd*, 2H, Ar-H, *J* = 8.5, 1.7 Hz), 7.46 (*s*, 1H, Ar-H), 7.14–7.16 (*m*, 2H, Ar-H), 7.12 (*dd*, 1H, Ar-H, *J* = 7.65, 1.7 Hz), 6.98 (*d*, 2H, Ar-H, *J* = 2.55 Hz), 6.92–6.91 (*m*, 2H, Ar-H), 6.89 (*d*, 1H, Ar-H, *J* = 1.7 Hz), 6.87 (*d*, 1H, Ar-H, *J* = 8.5 Hz), 6.84 (*d*, 1H, Ar-H, *J* = 7.65 Hz), 4.03 (*t*, 4H, 2OCH_2_, *J* = 6.8 Hz), 3.89 (*t*, 4H, 2OCH_2_, *J* = 6.8 Hz), 3.76 (*t*, 2H, NCH_2_, *J* = 6.8 Hz), 1.87 (*quint*, 4H, 2CH_2_, *J* = 6.8 Hz), 1.75 (*quint*, 4H, 2CH_2_, *J* = 7.65 Hz), 1.68 (*quint*, 4H, 2CH_2_, *J* = 6.8 Hz), 1.51 (*quint*, 2H, CH_2_, *J* = 7.65 Hz), 1.42–1.19 (*m*, 70H, 35CH_2_), 0.85–0.89 (*m*, 15H, 3CH_3_). ^13^C NMR; (213 M Hz, CDCl_3_) δ: 164.20, 162.40, 149.08, 146.76, 145.61, 145.16, 138.44, 137.30, 132.31, 130.92, 128.78, 127.15, 124.73, 122.63, 120.55, 119.50, 115.57, 113.67, 69.54, 69.24, 47.04, 31.92, 29.74, 29.70, 29.68, 29.66, 29.63, 29.60, 29.59, 29.55, 29.47, 29.40, 29.36, 29.32, 29.30, 27.21, 26.74, 26.67, 26.22, 26.06, 22.65, 14.12. IR; υ cm^−1^: C-H aliphatic 2923, 2853, C=N stretch 1733, C=C stretch 1568. HRMS (ESI): *m/z* calcd for *C*_79_*H*_124_*N*_3_*O*_4_*S*_2_ 1242.9033 [M+H]^+^, found 1242.9028.

## 3. Result and Discussion

### 3.1. Synthesis

**Scheme 1:**
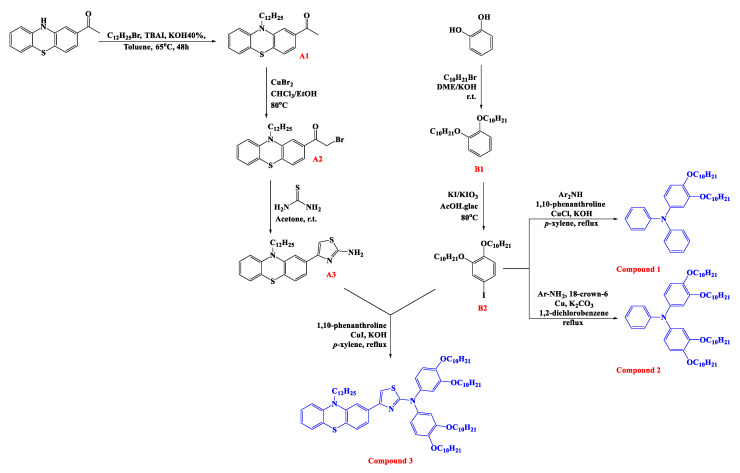
Synthesis of Compounds **1–3**.

Three triarylamine derivatives **Compounds 1–3** were designed and synthesized in good yields, as shown in Scheme 1. Thus, 2-acetylphenothiazine was *N*-alkylated via S_N_2 reaction using phase transfer catalyst (TBAI) followed by a side-chain bromination afforded **A2** in good yield. Double *O*-alkylation of catechol followed by an iodination reaction afforded the corresponding **B2** in good yield. Ullmann reaction of **B2** with diphenylamine or aniline was accomplished using 1,10-phenanthroline/CuCl/KOH or 18-crown-6/Cu/K_2_CO_3_ under reflux for 24 h to afford the corresponding **Compounds 1** and **2**, respectively. On the other hand, aminothiazole derivative **(A3)** was synthesized in an excellent yield through Hantzsch condensation of thiourea and *N*-dodecyl−2-(*α*-bromo)-acetylphenothiazine (**A2**) in acetone at room temperature overnight. Similarly, the Ullmann reaction of **B2** with **A3** was accomplished using 1,10-phenanthroline/CuI/KOH to afford the corresponding **Compound 3**. The long side-chain of decyloxy group was introduced to *para*- and *meta*-position of triphenylamine backbone to provide good solubility, thermal stability, and better film-forming in PSCs. The electron-acceptor thiazole ring was used as a linker between two good electron-donors, phenothiazine and diphenylamine, resulting in a donor–acceptor–donor conjugated small molecule that would have an impact on the charge transport characteristics compared with **Compounds 1** and **2**. The synthesized compounds were fully characterized, and their chemical structures were confirmed by NMR, IR and MS (S2–S33).

### 3.2. UV–Vis Absorption and Electrochemical Property

**Figure 1:**
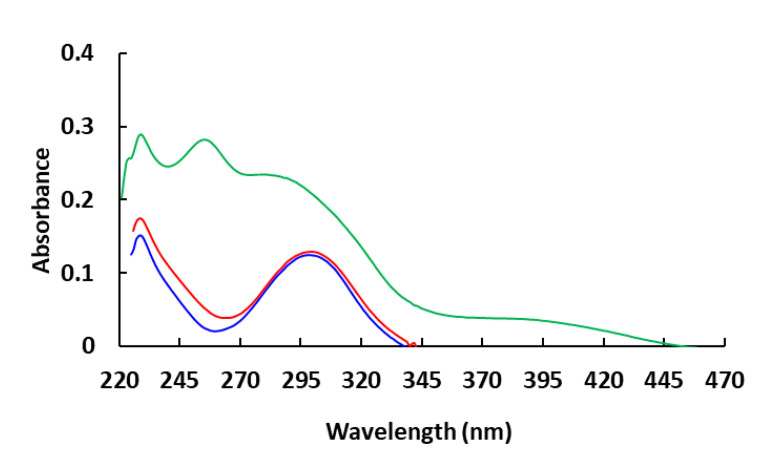
The experimental UV–Vis absorption spectra of the compounds in DCM solutions (10^−5^ mol/L).

As an essential characteristic of the synthesized molecules as HTMs, the UV–vis absorption profile of **Compounds 1–3** were measured in DCM, as shown in Figure 1 and Table S1. It is clear from Figure 1 that all compounds are absorbing at wavelengths below 300 nm or less (due to π–π* transition) except for **Compound 3** that shows ICT (intramolecular charge transfer) broadband at 292 nm owing to the thiazolyl moiety [43], which is overlapped with n–π* band nearby 395 nm. The major absorption peaks for these compounds are thus away enough from the maximum absorption wavelength of perovskite photosensitizer CH_3_NH_3_PbI_3−x_Cl_x_ (360 nm) [44], indicating that these compounds are transparent and potentially suitable as HTMs. The optical band gap energy (*E_g_*) for these compounds were obtained from the onset absorption as shown in Table 1.

**Figure 2:**
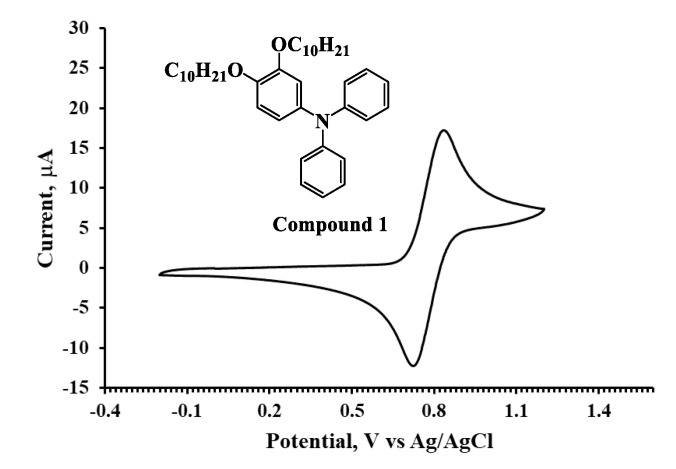
Cyclic voltammogram of **Compound 1** in DCM solution (10^−3^ M).

**Figure 3:**
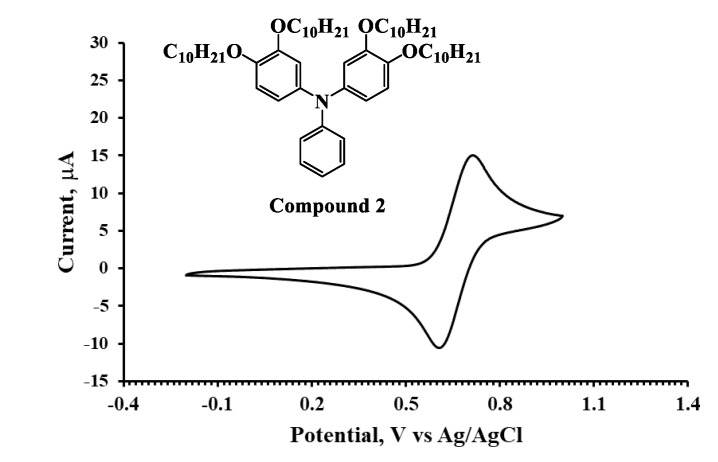
Cyclic voltammogram of **Compound 2** in DCM solution (10^−3^ M).

**Figure 4:**
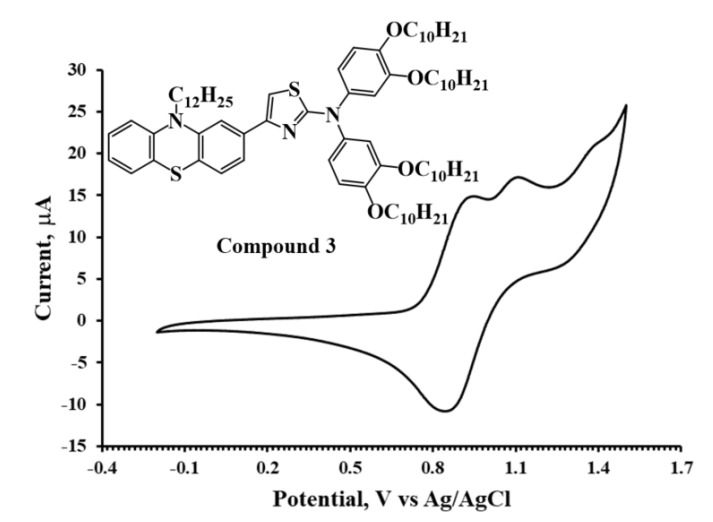
Cyclic voltammogram of **Compound 3** in DCM solution (10^−3^ M).

The HOMO energies for the synthesized compounds were measured by cyclic voltammetry from which the onset oxidation peaks shown in the voltammograms (Figure 2, Figure 3 and Figure 4) were used to calculate the HOMO energies. The LUMO energies were then obtained by the summation of *E_g_* with the HOMO energy as shown in Table 1.

### 3.3. Computational Investigation

#### 3.3.1. Ground-State Geometries and Frontier Molecular Orbitals

**Figure 5:**
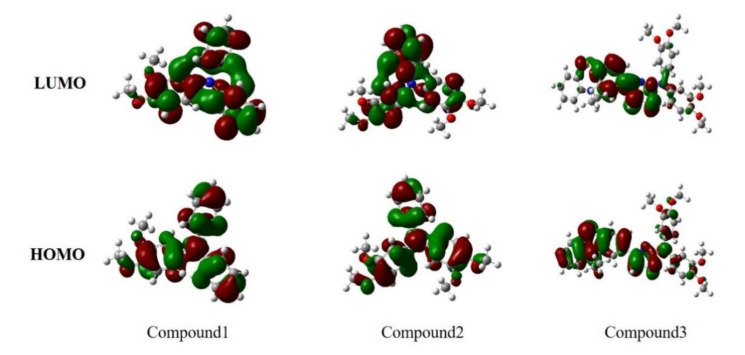
The molecular orbitals HOMO and LUMO of the three original molecules.

As an approximation for easy calculation, it is generally accepted for an organic molecule that replacement of a long chain (in our case -C_12_H_25_ and -C_10_H_21_) with short ones such as -C_2_H_5_ and -CH_3_, respectively, during the structural optimization will have a marginal effect on the electronic properties [47]. Thus, DFT/B3LYP/6-31G(d) has been used to optimize the three compounds after being simplified with short chains. The optimized structures and parameters were shown in Supplementary Materials: Figure S1 and Table S2. The bond lengths between nitrogen atoms and benzene rings of compounds have a slight difference. Moreover, **Compounds 1** and **2** have slightly different dihedral angles, which indicates that the length of hydrocarbon chains had little influence on the planarity. However, it had obviously changed the planarity when phenothiazine was introduced to diphenylamine moiety through thiazole ring. The HOMO and LUMO indicate the distribution of the electronic density, respectively, which contributed to electron transition. It is shown in Figure 5 that the electronic density of the HOMO of both **Compounds 1** and **2** are mainly distributed over the whole molecule except the side alkyl groups. The LUMO’s electronic density of **Compounds 1** and **2** are mainly distributed over diphenylamine moiety and phenylamine moiety, respectively. In **Compound 3**, however, the electronic density distribution in the HOMO level is mainly distributed on the thiazolylphenothiazine moiety with a little distribution on diarylamine moiety. The energy of LUMO of **Compound 3** shows that the electronic density distribution has moved toward the center of the molecule.

**Figure 6:**
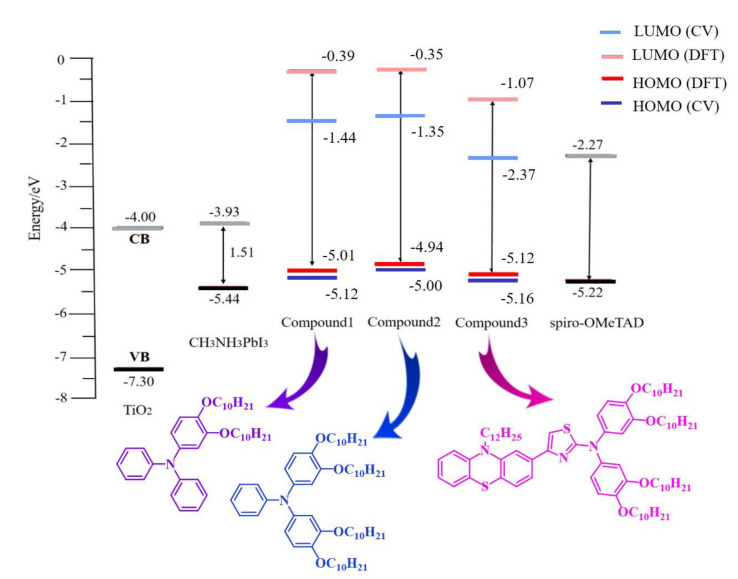
Energy level diagram of the investigated hole transporting materials.

The frontier molecular orbitals energy levels (HOMO and LUMO) were calculated and the results are listed in Table 2 and Figure 6. As shown in Table 2, the HOMO energy levels were in the increasing order **Compound 3** < **Compound 1** < **Compound 2**. The energy gap of HTMs as estimated by ∆_H−L_ = E_LUMO_ − E_HOMO_ reflects that **Compound 3** revealed the lowest bandgap among the three molecules. These results indicate that **Compound 3** is the most energy-stabilized molecule compared to **Compounds 1** and **2,** owing to its lower HOMO and bandgap energies, in a good correlation with the extended conjugation caused by the thiazole ring that acts as an internal auxiliary acceptor [43]. The energy levels of HTMs and perovskite (CH_3_NH_3_PbI_3_), and the reference Spiro-OMeTAD, are shown in Figure 6. As shown in Figure 6, the experimental and computational values of the HOMO energies were in good agreement. The HOMO energy levels for **Compounds 1–3** were higher than the valence band (VB) of the perovskite film, thus indicating their suitability for hole injection. At the same time, their LUMO energy levels were all higher than that of the conduction band of perovskite film that benefits the inhabitation of the electron back in the perovskite system.

#### 3.3.2. Density of States (DOS) and Frontier Molecular Orbitals

**Figure 7:**
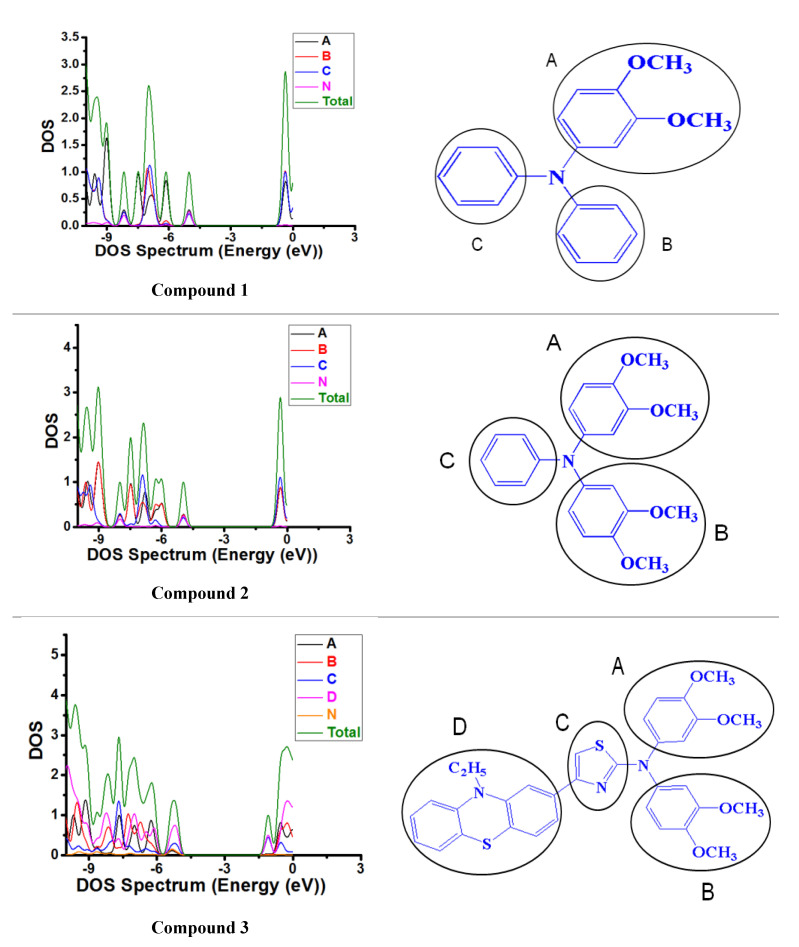
Partial density of states graphs of three compounds.

In order to get a clear view of the roles of each part among the compounds. The DOS and partial density of states (PDOS) were plotted under the GaussSum software. Additionally, the DFT with B3LYP functional at the 6–31G(d) basis set was used to calculate the DOS and PDOS of **Compounds 1–3**. The PDOS are shown in Figure 7, and the contribution of every unit for DOS are listed in Table S3. As shown in Figure 7, when the B part (phenyl) of **Compound 1** is replaced by dimethoxyphenyl to produce **Compound 2**, the contribution of the B part of the molecule to HOMO orbital was slightly increased. When we replaced the C part (phenyl) with the thiazole-based on **Compound 2** and introduced the D part (10-ethyl-10*H*-phenothiazine) at the end of the C part, the contribution of the C part to the HOMO orbital remained unchanged; the contribution of the A part, B part and N to the HOMO orbital was greatly reduced, and the newly introduced D part provides great contribution for the HOMO orbital. The HOMO level of the molecule was also decreased, which is favorable for hole injection. Since the LUMO of **Compounds 1–3** were all much higher than the conduction band (CB) of perovskite film, we did not analyze the LUMO orbitals of **Compounds 1–3** in detail here. To sum up, the introduction of dimethoxyphenyl will increase the HOMO energy levels of the molecules, which will have a very adverse effect on the hole injection. However, the introduction of 10-ethyl-10*H*-phenothiazine will further reduce the HOMO energy levels of the molecules, which will have a very favorable impact on the hole injection.

#### 3.3.3. Ionization Potentials (IP), Electron Affinities (EA) and Absolute Hardness

As shown in Table 3, the IP and EA values indicate that **Compound 2** had both the lowest ionization potential and electron affinity, indicating its suitability as the best hole transfer molecule among the studied compounds. It has been reported that the value of chemical hardness has an impact on the intramolecular charge transfer in such a way that a higher hardness value would help to resist the intramolecular charge transfer and the absolute hardness *η* means the ability of energy to resist electron changes [47,48]. Among three molecules, **Compound 2** has both the lowest IP, EA and relatively higher value of *η*, and thus the overall results suggest **Compound 2** to be the suitable HTM.

#### 3.3.4. Absorption Spectra

**Figure 8:**
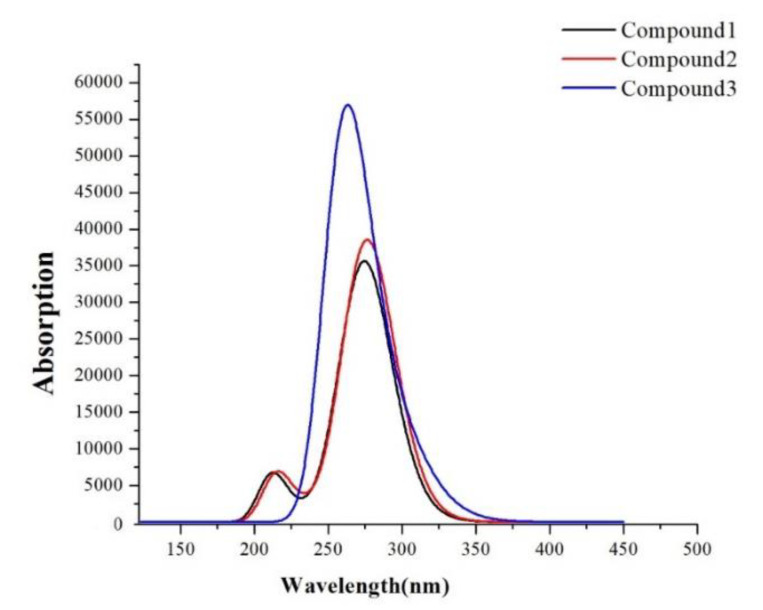
The computed UV–Vis absorption spectra of the compounds.

The absorption spectra are shown in Figure 8, and transition information are listed in Table 4. Thereinto, the absorption wavelength is an important parameter to describe optical response. As shown in Table 4, the values of S1 states of them were calculated to be 277.67 nm (for **Compound 1**), 278.14 nm (for **Compound 2**) and 302.71 nm (for **Compound 3**), respectively. From the simulated spectra, it can be found that **Compound 2** made a little red-shifted spectrum compared to **Compound 1**, and **Compound 3** made a blue-shifted spectrum about 10 nm. We can see further from Table 4, the order of the absorption peak was **Compound 2** > **Compound 1** > **Compound 3**, and the absorption peaks **of Compounds 1–3** were all in the range of 250–350 nm (Figure 1 and Table S1 in Supplementary Materials). It further proves the accuracy of the calculated data on absorption spectra. Absorption spectra for the three compounds reside in the non-visible region, which benefits optical transparency.

#### 3.3.5. Fluorescence Lifetime

The fluorescence lifetime *τ*_1_ and excited-state lifetime *τ*_2_ of the molecules both reflect the time that the exciton stays in the excited state. Previous reports on carbazole substituted NP based derivative as HTM showed that the short excitation lifetime could depress unfavorable relaxation of the excited state [36]. The fluorescence energy and fluorescence lifetime *τ*_1_ are shown in Table 5, and the vertical excitation energy and excited-state lifetime *τ*_2_ and the hardness ƞ are shown in Table S4. From the point of view of the lifetime, it can be found that **Compound 3** had the significantly longer *τ*_1_ and *τ*_2_ rather than other compounds, which could increase its relaxation and this process is disadvantageous for the excited molecules to the ground state. However, the fact that **Compounds 1** and **2** had higher hardness ƞ and a smaller lifetime could promote the two compounds to have good HTM performance in comparison with **Compound 3**.

#### 3.3.6. Charge Transport and Hole Mobility

Hole transport is a critical parameter for estimation of HTMs performance and well performance of HTMs will promote the improvement of short-circuit currents. Table 6 and Table 7 show the calculated hole reorganization energies and hole mobilities, which show the order of the hole mobilities as **Compound 2** > **Compound 3** > **Compound 1**. We can also see from Table 6 that **Compound 3** had lower hole reorganization energy, which was conducive to the improvement of hole mobilities; but **Compound 3** had small hole transfer integrals, which was very unfavorable for further development of hole mobility. It is hoped that the addition of -OCH_3_ can improve the hole transfer integrals and can further improve the hole transport in the design of the triphenylamine system.

## 4. Conclusions

Three novel triarylamine **Compounds 1–3** were designed and synthesized for perovskite solar cells. Optical absorption and computational investigation indicated the suitability of these compounds as HTMs as they are transparent in the absorption region of the perovskite photosensitizer. The experimental and computational values of HOMO energies were in good agreement and the values were suited well with the perovskite photosensitizer. Additionally, the computed hole transfer mobility was the best for **Compound 2** among the three compounds. Future studies will focus on the film forming ability of these compounds and their use for device fabrication and application as HTMs for the perovskite solar cell and the outcome will be reported in a future article.

## Figures and Tables

**Table 1:** Summary of energy levels and *E_g_* of **Compounds 1**–**3**.

Molecule	Λ ^a^ (nm)	*E_g_*^b^ (eV)	*E_ox_*^c^ (V)	*E_ox_* (Ferrocene) ^c^ (V)	HOMO ^d^ (eV)	LUMO ^e^ (eV)
**Compound 1**	337	3.68	0.72	0.40	−5.12	−1.44
**Compound 2**	340	3.65	0.60	0.40	−5.00	−1.35
**Compound 3**	445	2.79	0.76	0.40	−5.16	−2.37

^a^ λ_onest_ Measured by UV–Vis absorption spectra (Figure 1); ^b^ The optical band gap was estimated from the onset wavelength of absorption using the equation *E_g_* = 1240/λ_onset_; ^c^ oxidation potentials measured by cyclic voltammetry (Figure 2, Figure 3 and Figure 4); ^d^ HOMO = −(*E_ox_* + 4.80 − *E_ox_* (Ferrocene)) eV [45,46]; ^e^ The LUMO energy level was calculated using the equation *E*_LUMO_ = *E*_HOMO_ + *E_g_*.

**Table 2:** Calculated energy levels of frontier molecular orbital (HOMO and LUMO), and the energy gaps Δ_L-H_ of the three studied molecules.

Molecules	HOMO (eV)	LUMO (eV)	Δ_L-H_ (eV)
**Compound 1**	−5.007438148	−0.387762165	4.619675983
**Compound 2**	−4.938049129	−0.353203712	4.584845416
**Compound 3**	−5.125263423	−1.071039917	4.054223506

**Table 3:** The ionization potential IP (eV), electron affinities EA (eV) and absolute hardness *η* (eV).

Molecules	Compound 1	Compound 2	Compound 3
IP	4.826496076	4.759131584	4.920280097
EA	0.524809559	0.515998514	1.29218136
*η*	2.150843259	2.121566535	1.814049369

**Table 4:** The vertical excitation energy (eV), absorption peak (nm), configuration interaction (CI) coefficient and oscillator strengths (*f*) of investigated molecules.

Molecules	State	Energy	λ	*f*	CI
**Compound 1**	S1	4.4652	277.67	0.2767	H-L/0.66564
	S2	4.5111	274.84	0.3910	H-L + 1/0.66581
	S3	4.5623	271.76	0.2050	H-L + 2/0.65691
	S4	4.9924	248.34	0.0315	H-L + 4/0.48494
	S5	5.0173	247.11	0.0321	H-L + 3/0.53146
	S6	5.8306	212.64	0.1661	H-L + 2/0.38636
**Compound 2**	S1	4.4577	278.14	0.3412	H-L/0.64282
	S2	4.4771	276.93	0.4976	H-L + 1/0.65286
	S3	4.5514	272.41	0.1049	H-L + 2/0.65475
	S4	4.9754	249.19	0.0358	H-L + 3/0.44687
	S5	4.9942	248.26	0.0271	H-L + 4/0.42356
	S6	5.7443	215.84	0.1691	H − 1-L + 2/0.50837
**Compound 3**	S1	4.0959	302.71	0.1619	H-L/0.57662
	S2	4.3452	285.33	0.1596	H − 1-L/0.48943
	S3	4.5086	274.99	0.0480	H-L + 3/0.39172
	S4	4.6656	265.74	0.1675	H-L + 1/0.43774
	S5	4.7392	261.61	0.8023	H − 1-L + 2/0.36926
	S6	4.7891	258.89	0.3343	H-L + 5/0.42187

**Table 5:** The fluorescence energy (eV), fluorescence emission peak (nm), oscillator strengths (*f*), electron transitions and fluorescence lifetime *τ*_1_ (ns) of investigated molecules.

Molecules	State	Energy	λ	*f*	CI	*τ*_1_
**Compound 1**	S1	3.8032	326.00	0.2075	H-L/0.69026	7.6785
**Compound 2**	S1	3.8016	326.14	0.2227	H-L/0.68822	7.1604
**Compound 3**	S1	2.9393	421.82	0.0355	H-L/0.64191	75.141

**Table 6:** The hole reorganization energies *λ_h_* (eV) and electron reorganization energies *λ_e_* (eV) of three investigated molecules.

Molecules	Compound 1	Compound 2	Compound 3
*λ_h_*	0.483489079	0.495456644	0.435409291
*λ_e_*	0.319682013	0.375718408	0.531043686

**Table 7:** The transfer integrals *V* (eV), *r* (Å) is centroid to centroid distance, hopping rates *k* (s^−1^) and mobilities *µ* (cm^2^ V^−1^s^−1^) of molecules.

Molecules	Compound 1	Compound 2	Compound 3
*r*	5.36846	4.87962	4.90008
*V_h_*	0.00217691	0.06802845	0.024898413
*V_e_*	0.001496626	0.030204632	0.02938829
*k_h_* (×10^11^)	0.0106386	9.14143	2.33463
*k_e_* (×10^11^)	0.0301456	6.58764	1.16805
*µ_h_*	0.000059301	0.042098	0.010842
*µ_e_*	0.00016803	0.030337	0.0054243

## Data Availability

The data presented in this study are available in Supplementary Materials here.

## Supplementary Materials

The following are available online at https://www.mdpi.com/article/10.3390/ma14113128/s1, the supplementary file (Figures S1–S33 and Tables S1–S4) contains the NMR, ATR-FTIR, HRMS charts and others for the synthesized compounds.
