# Supplementary material for: Novel Triarylamine-Based Hole Transport Materials: Synthesis, Characterization and Computational Investigation
Source: Materials (Basel). 2021 Jun 7;14(11):3128. doi: 10.3390/ma14113128 (PMC8200960; doi:10.3390/ma14113128)
Supplement: Supplementary file 1 [file materials-14-03128-s001.zip › materials-1208202-supplementary.pdf]

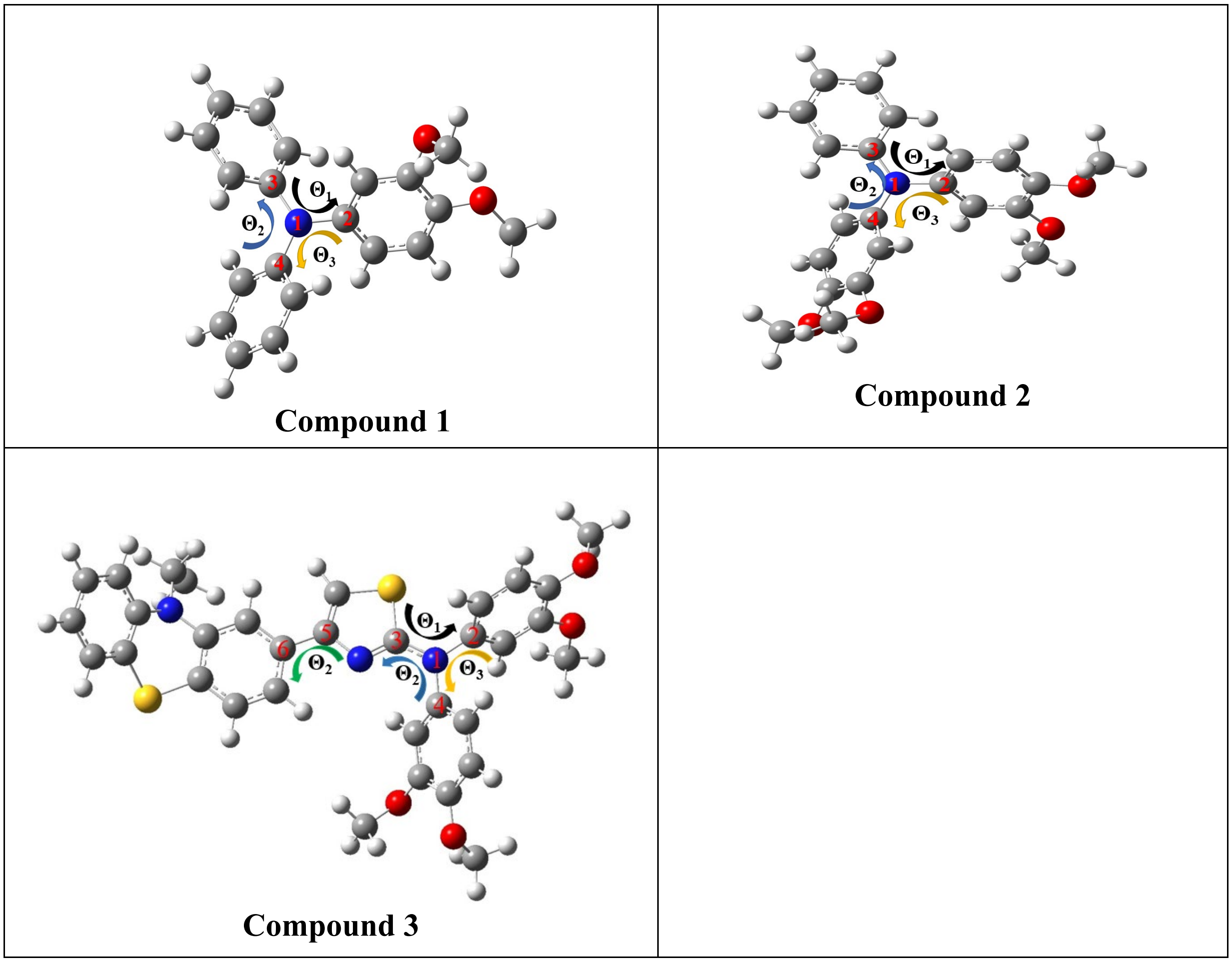

Figure S1. The chemical construction of the investigated molecules.

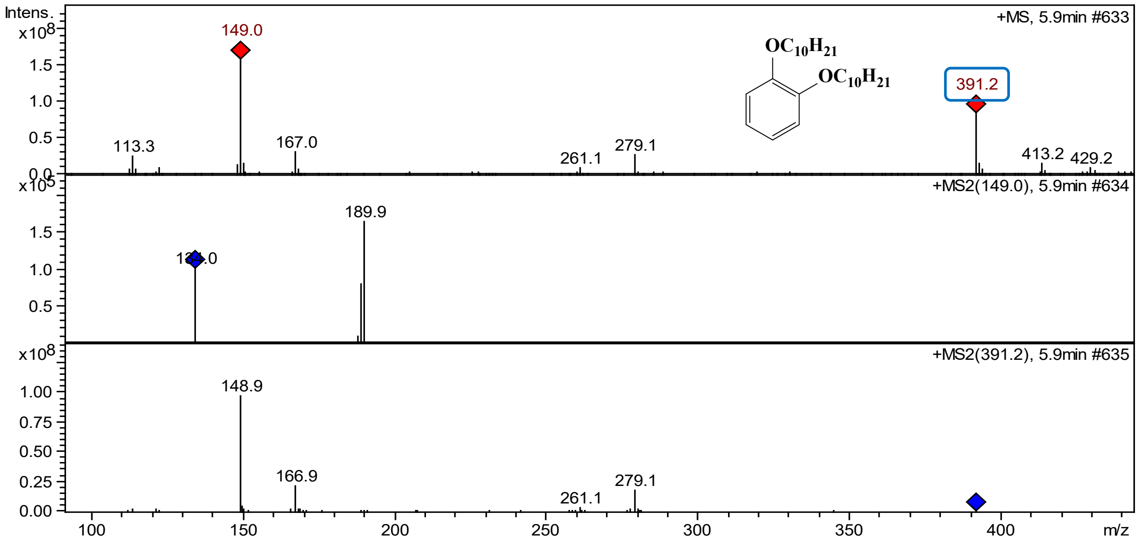

Figure S2. Mass spectrum of **B1.**

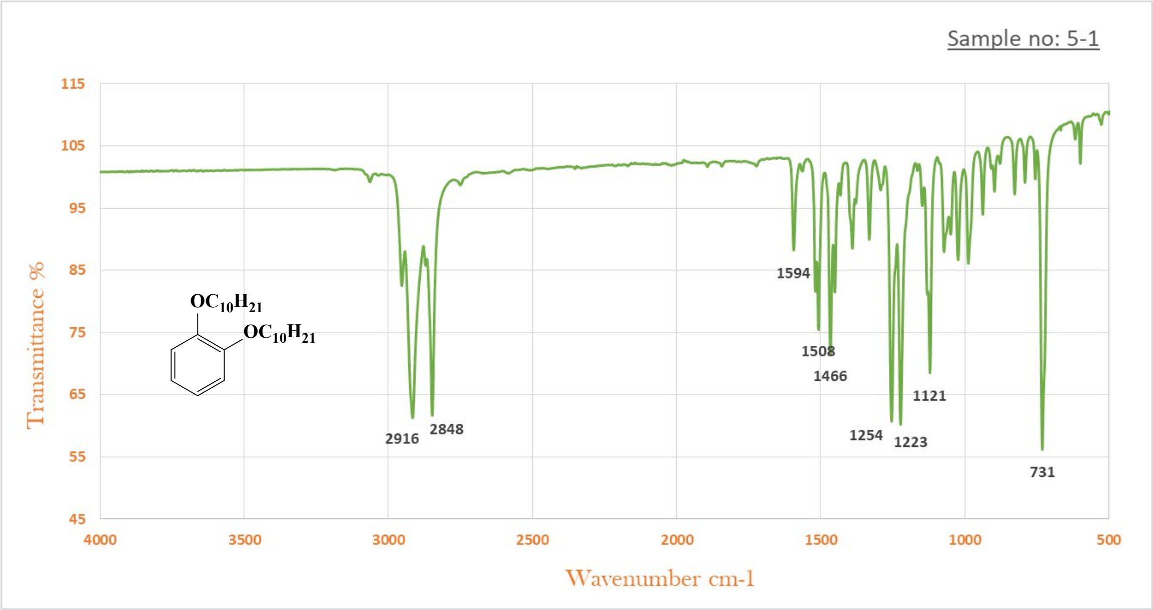

Figure S3. IR spectrum of **B1.**

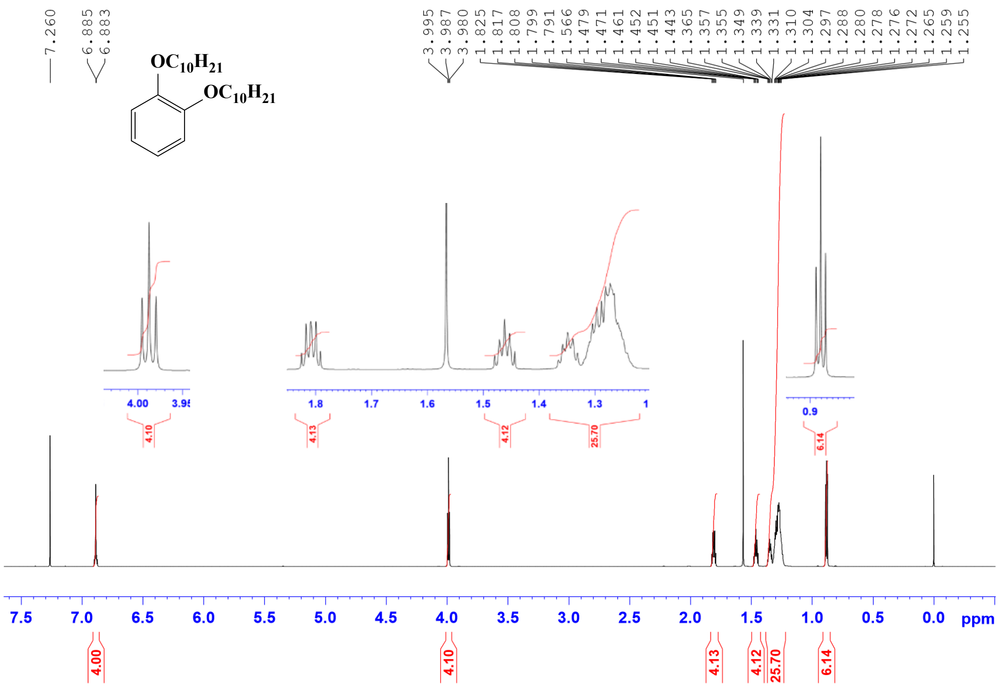

Figure S4. ^{1}H NMR ($CDCl_3$) spectrum of **B1.**

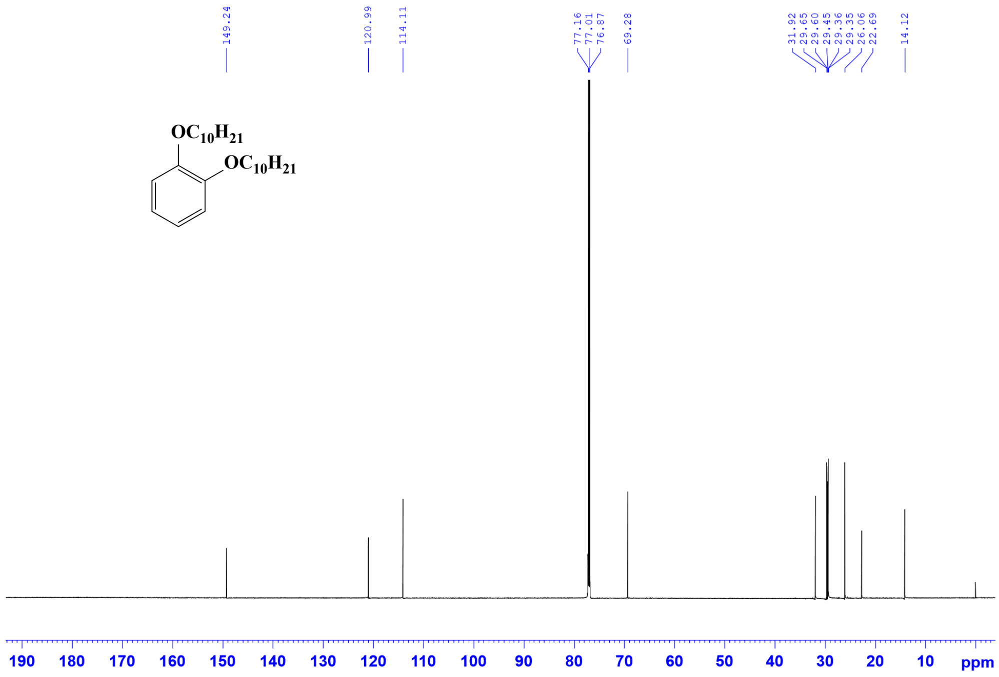

Figure S5. ^{13}C NMR ($CDCl_3$) spectrum of **B1.**

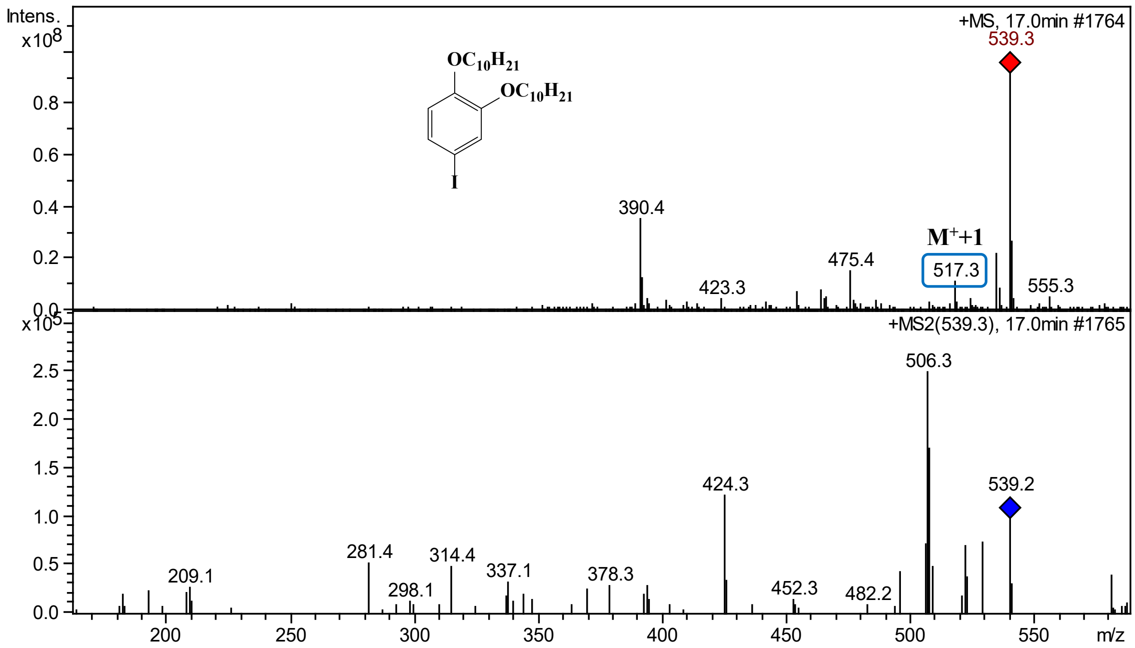

Figure S6. Mass spectrum of **B2.**

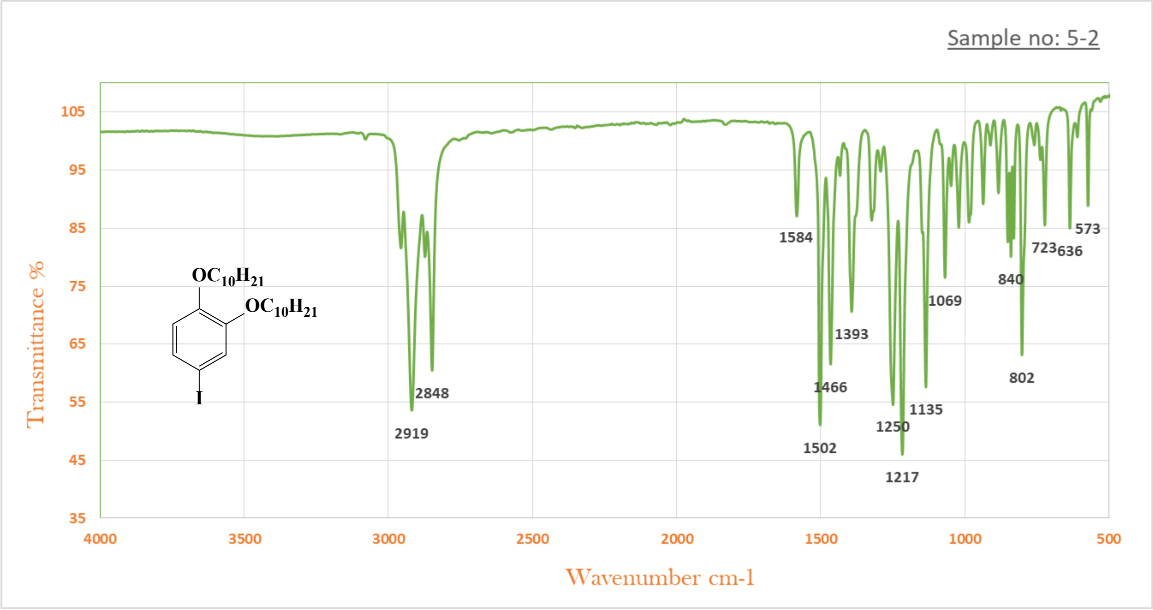

Figure S7. IR spectrum of **B2.**

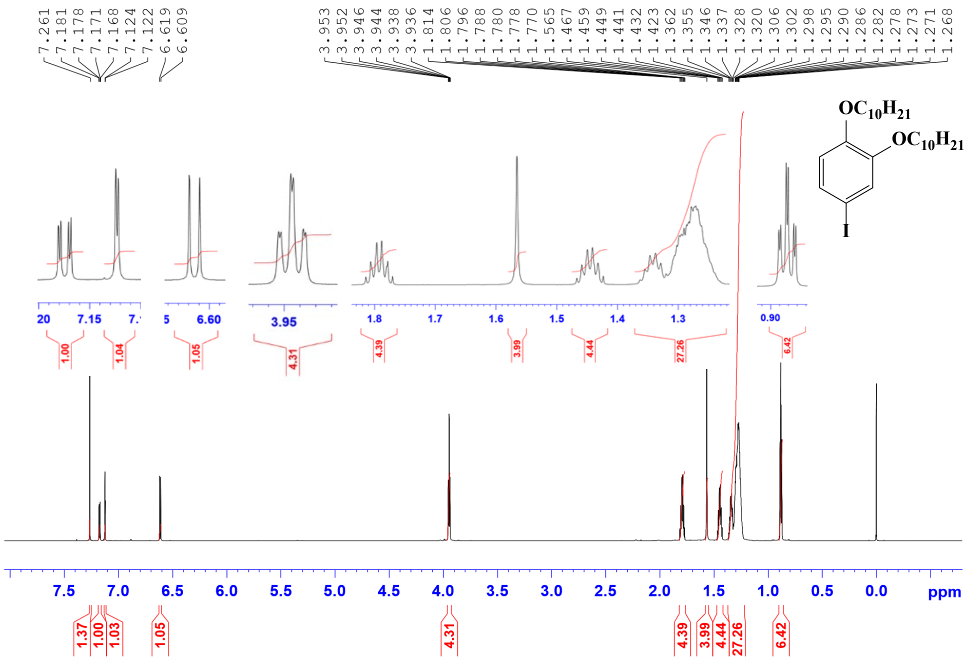

Figure S8. ^{1}H NMR ($CDCl_3$) spectrum of **B2.**

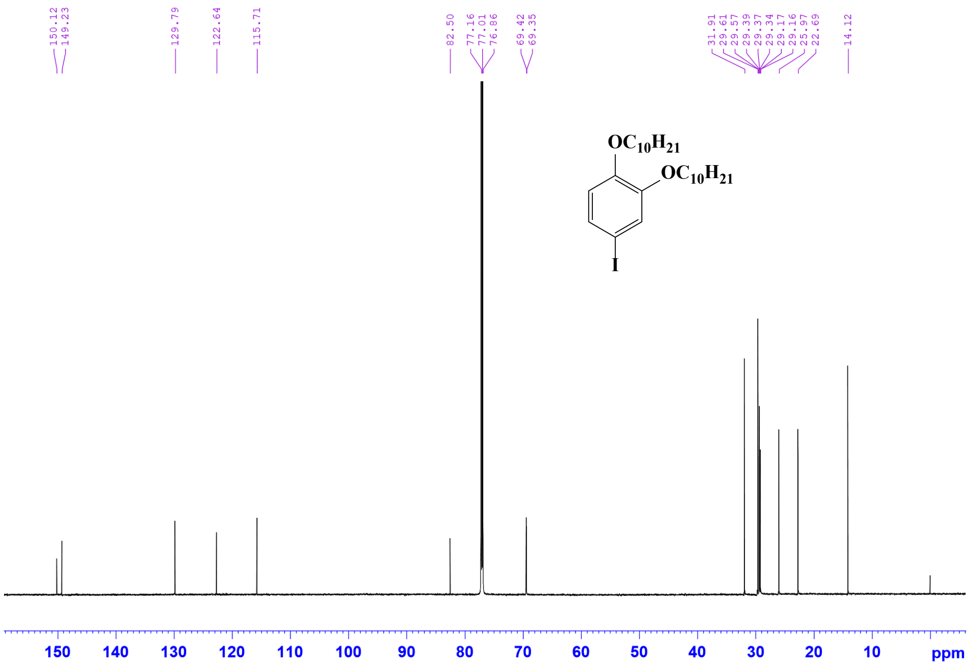

Figure S9. ^{13}C NMR ($CDCl_3$) spectrum of **B2.**

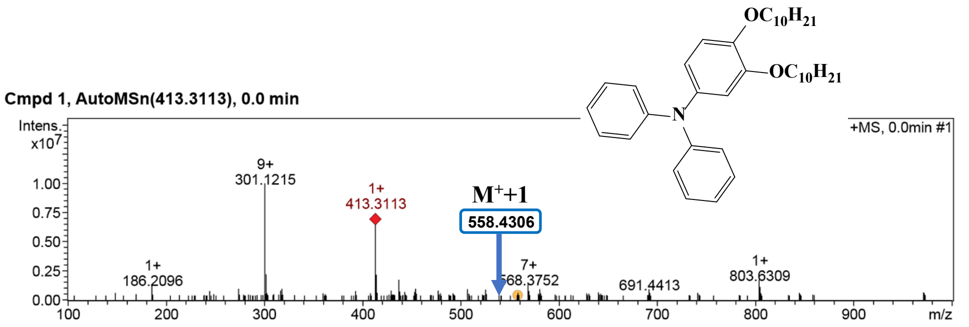

Meas. m/z	#	Ion Formula	m/z	err [ppm]	Mean err [ppm]	rdb	N-Rule	e⁻ Conf	mSigma	Std I	Std Mean m/z	Std I VarNorm	Std m/z Diff	Std Comb Dev
557.853786	1	C38H56NO2	558.430556	1032.8	1055.2	11.5	ok	even	292.6	315.0	n.a.	n.a.	n.a.	n.a.

Figure S10. High resolution mass spectrum of **compound 1.**

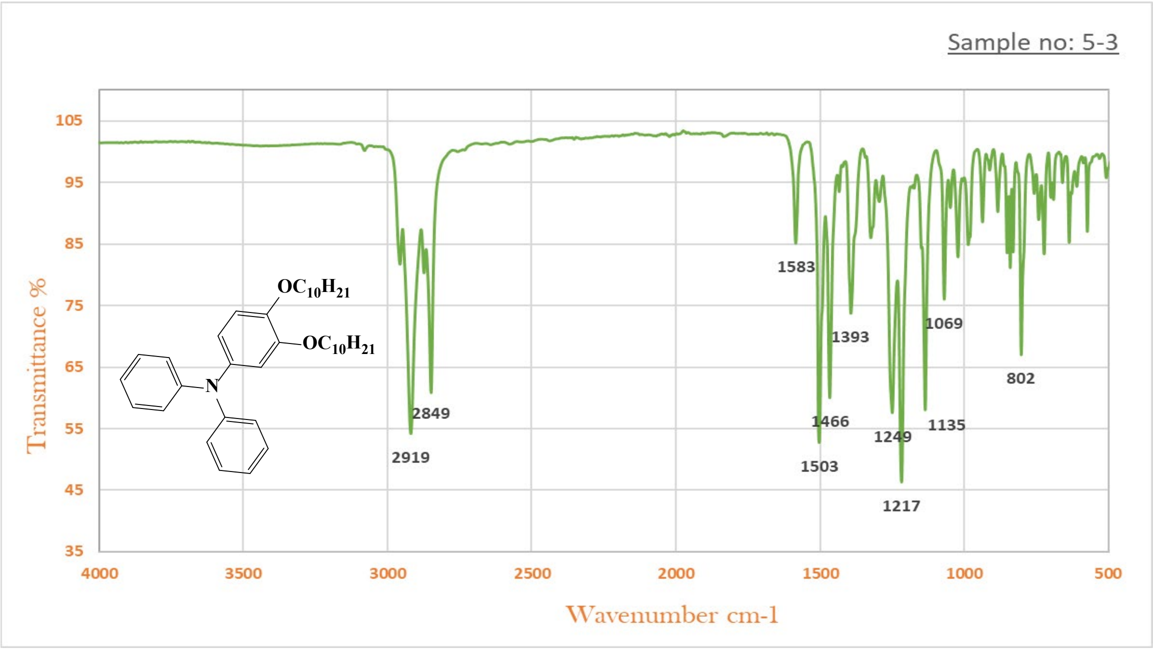

Figure S11. IR spectrum of **compound 1.**

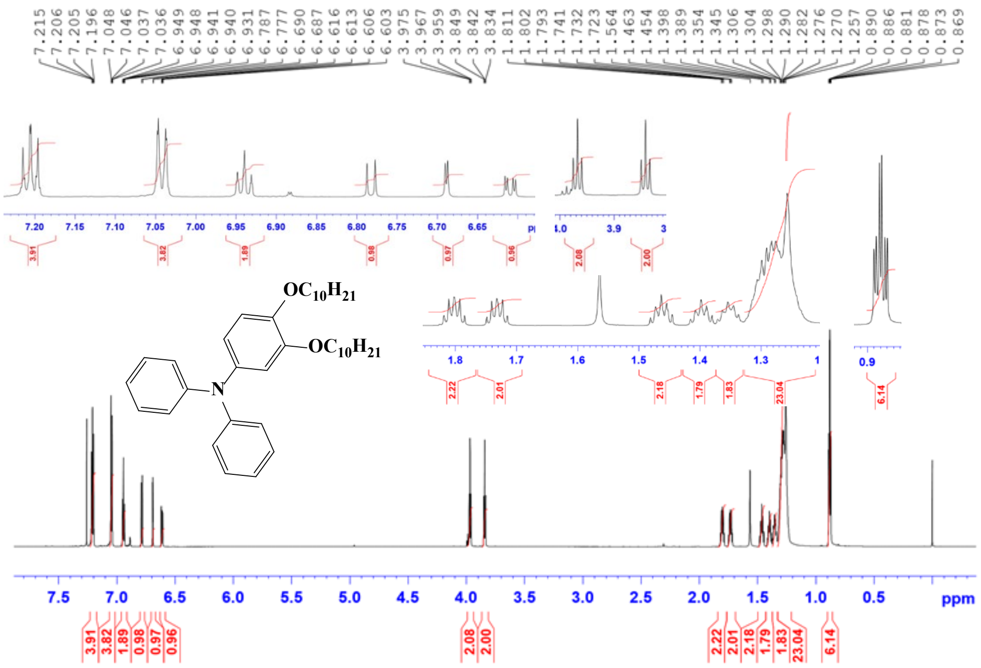

Figure S12. ^{1}H NMR ($CDCl_3$) spectrum of **compound 1.**

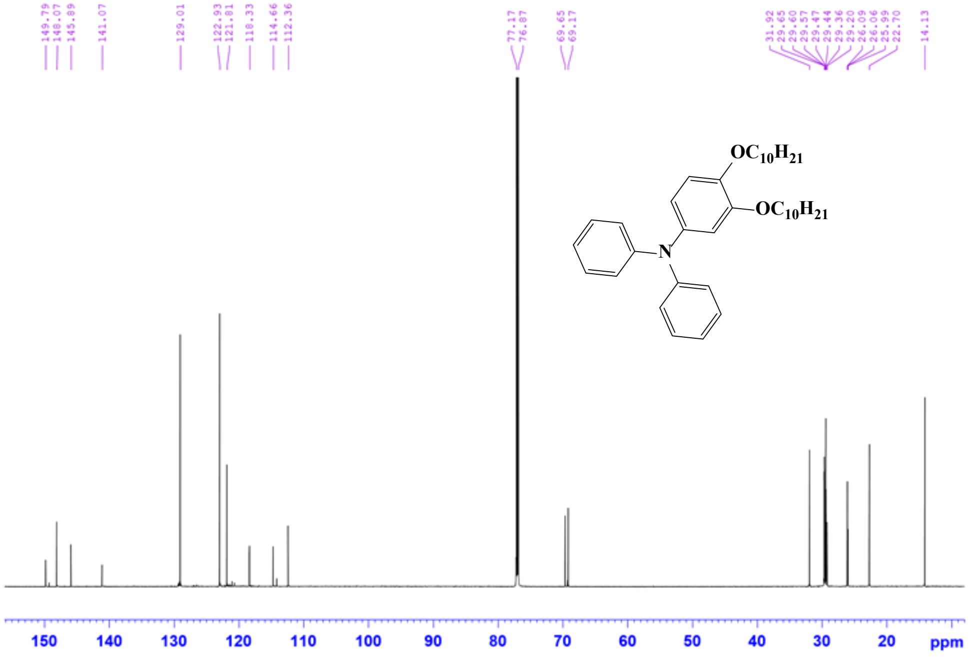

Figure S13. ^{13}C NMR ($CDCl_3$) spectrum of **compound 1.**

.

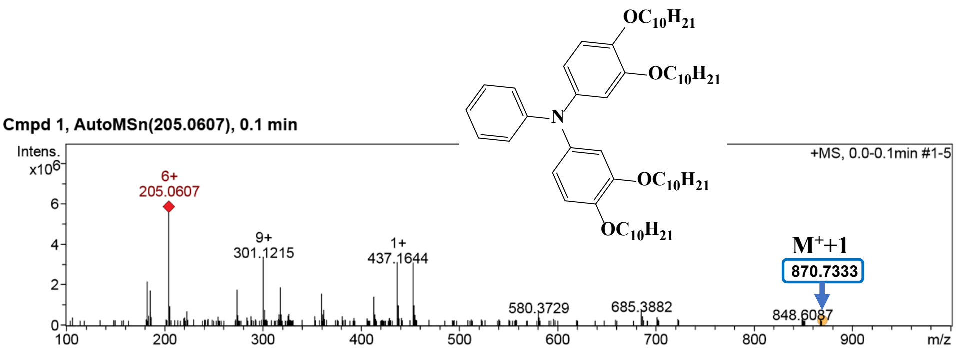

Meas. m/z	#	Ion Formula	m/z	err [ppm]	Mean err [ppm]	rdb	N-Rule	e⁻ Conf	mSigma	Std I	Std Mean m/z	Std I VarNorm	Std m/z Diff	Std Comb Dev
869.313513	1	C58H96NO4	870.733387	1630.7	1631.8	11.5	ok	even	162.8	189.8	n.a.	n.a.	n.a.	n.a.

Figure S14. High resolution mass spectrum of **compound 2.**

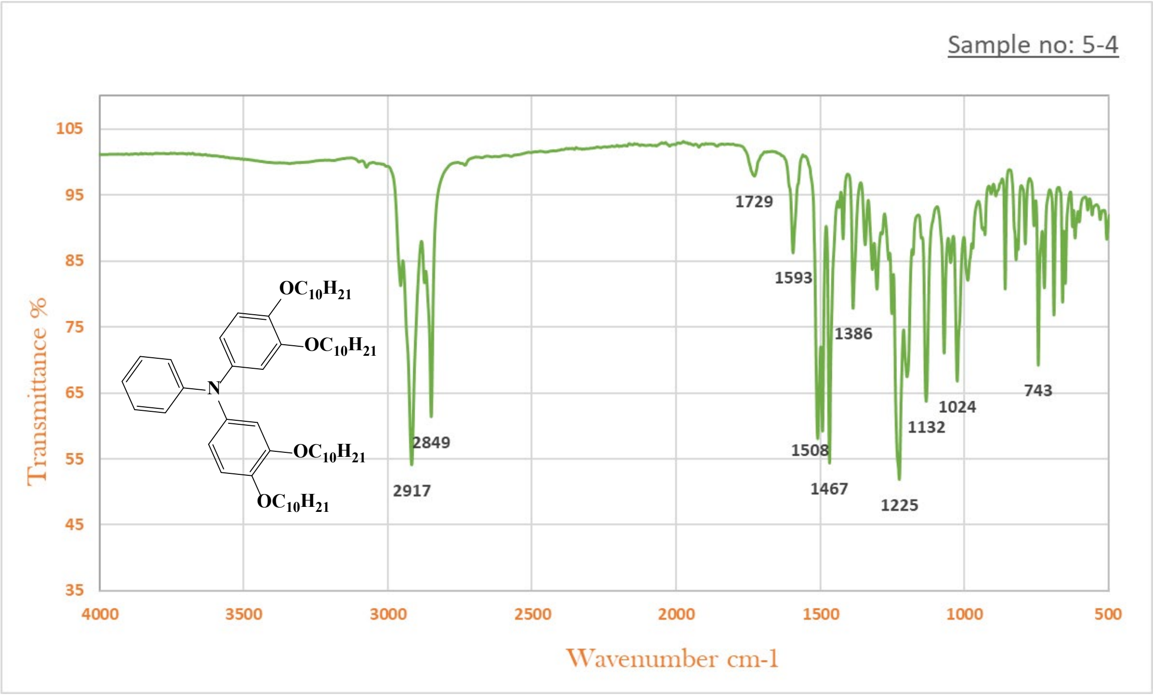

Figure S15. IR spectrum of **compound 2.**

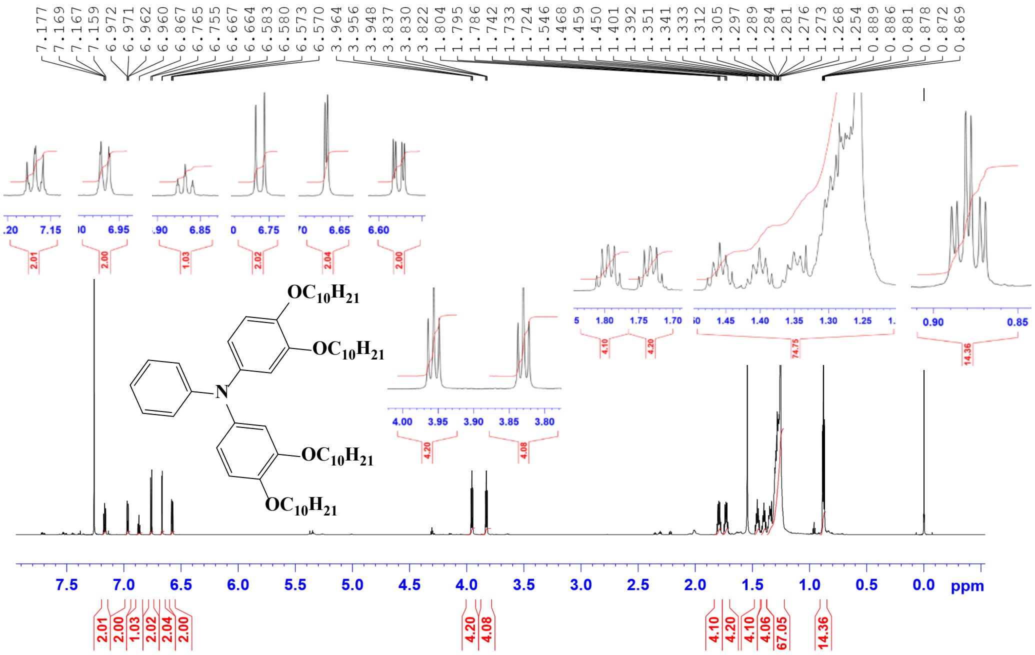

Figure S16. ^{1}H NMR ($CDCl_3$) spectrum of **compound 2.**

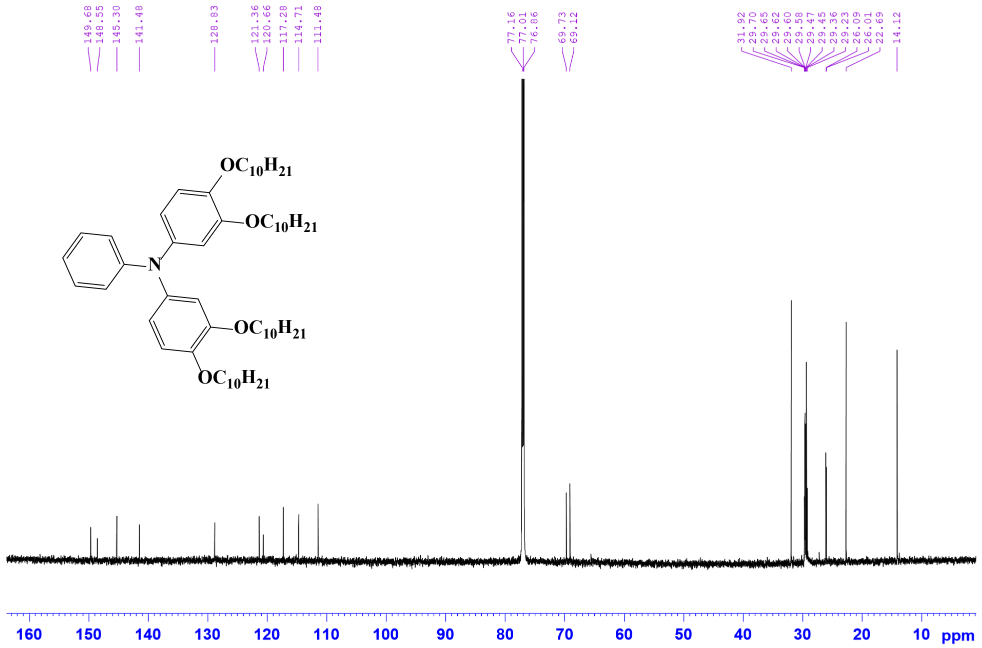

Figure S17. ^{13}C NMR ($CDCl_3$) spectrum of **compound 2.**

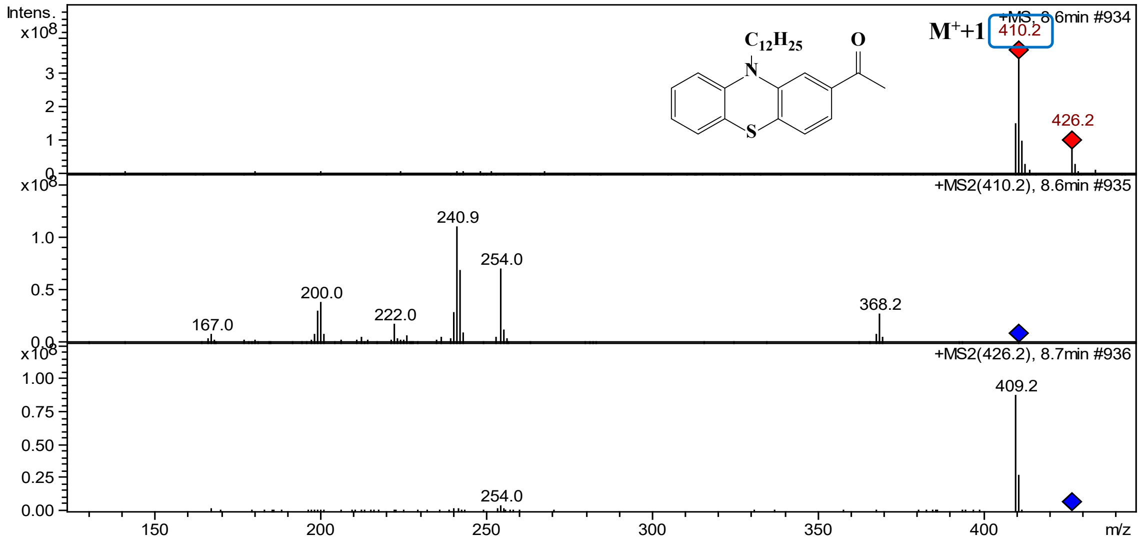

Figure S18. High resolution mass spectrum of **A1.**

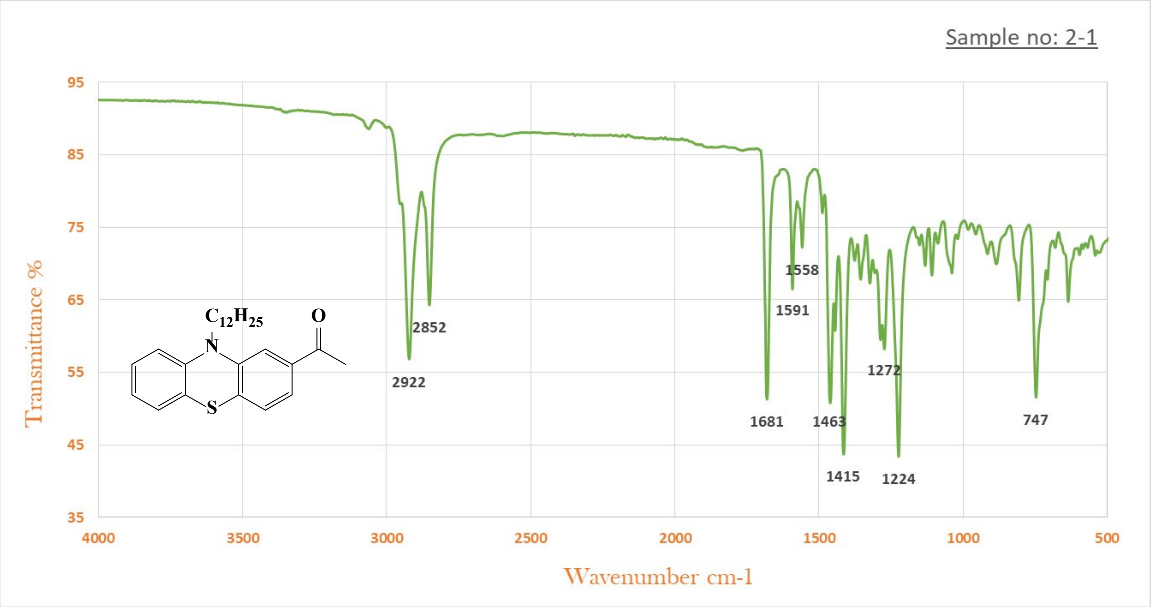

Figure S19. IR spectrum of **A1.**

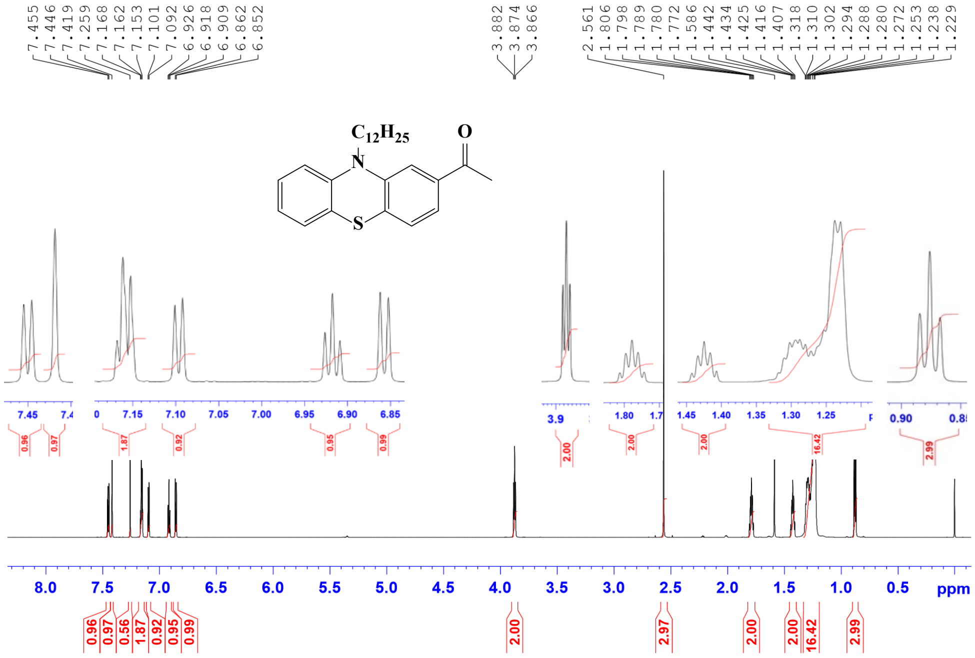

Figure S20. ^{1}H NMR ($CDCl_3$) spectrum of **A1.**

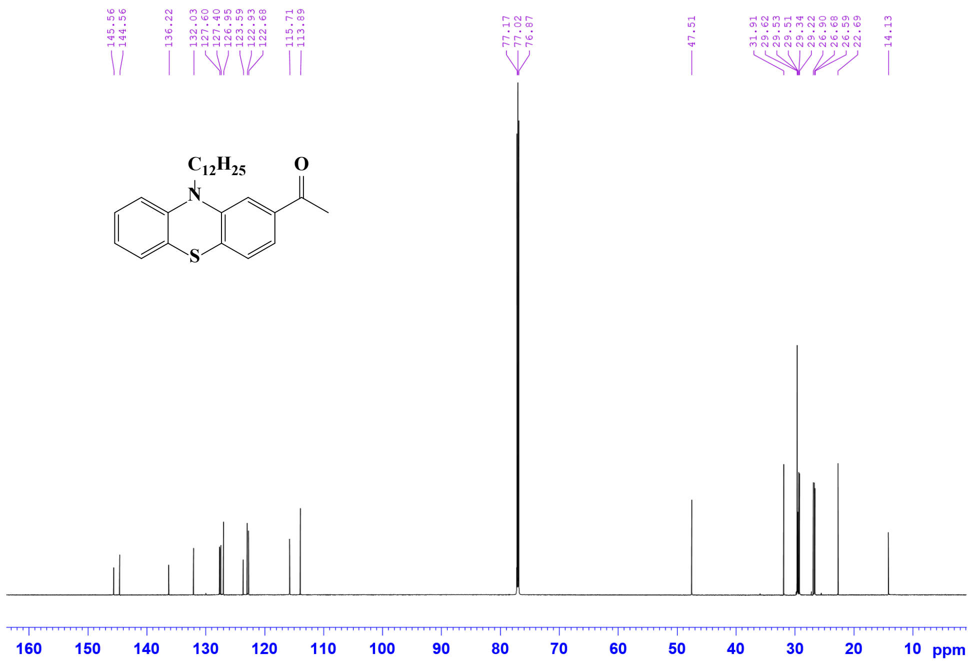

Figure S21. ^{13}C NMR ($CDCl_3$) spectrum of **A1.**

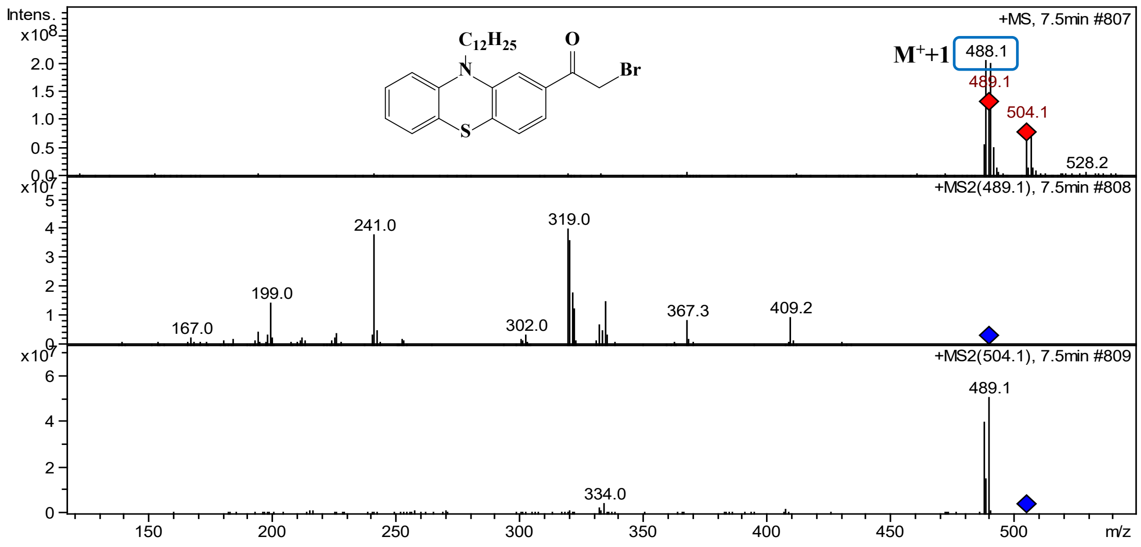

Figure S22. Mass spectrum of **A2.**

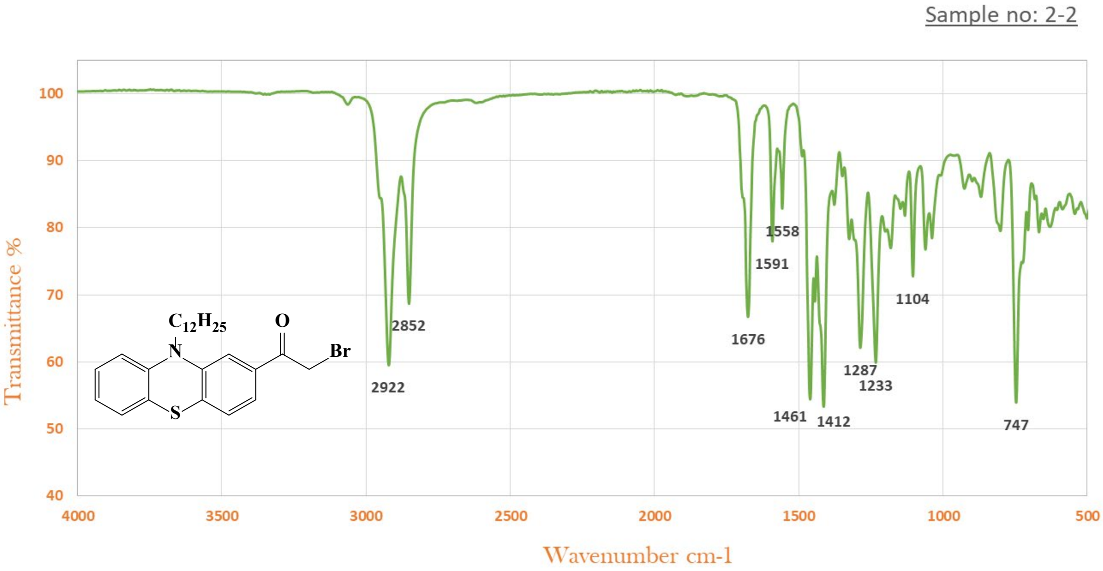

Figure S23. IR spectrum of **A2.**

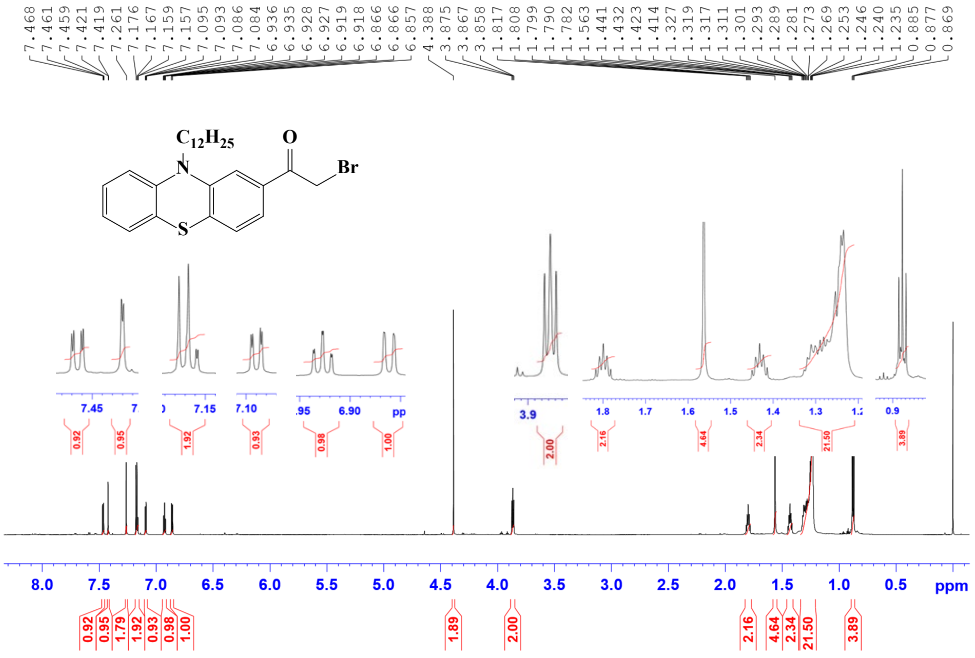

Figure S24. ^{1}H NMR ($CDCl_3$) spectrum of **A2.**

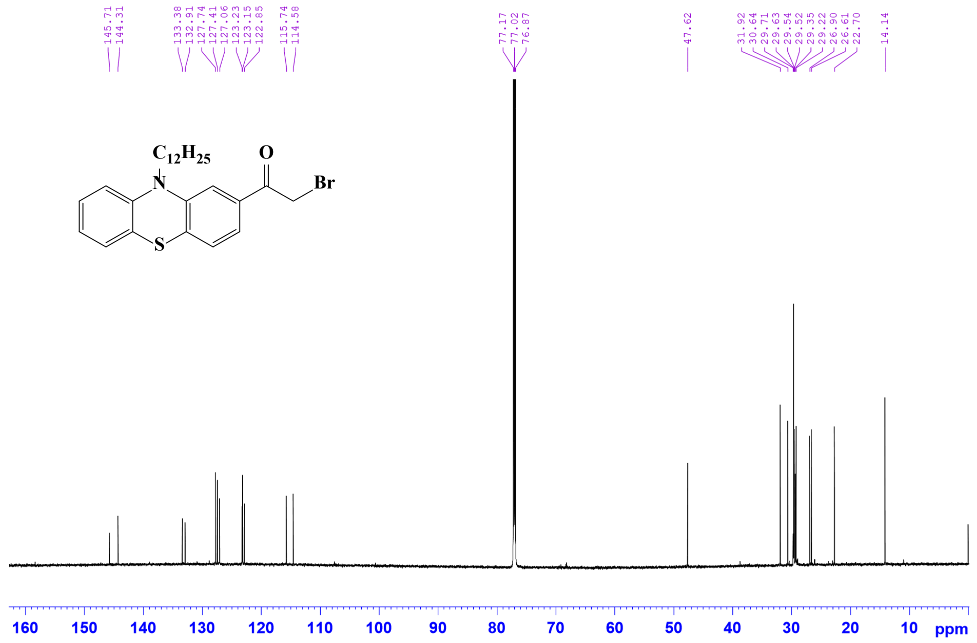

Figure S25. ^{13}C NMR ($CDCl_3$) spectrum of **A2.**

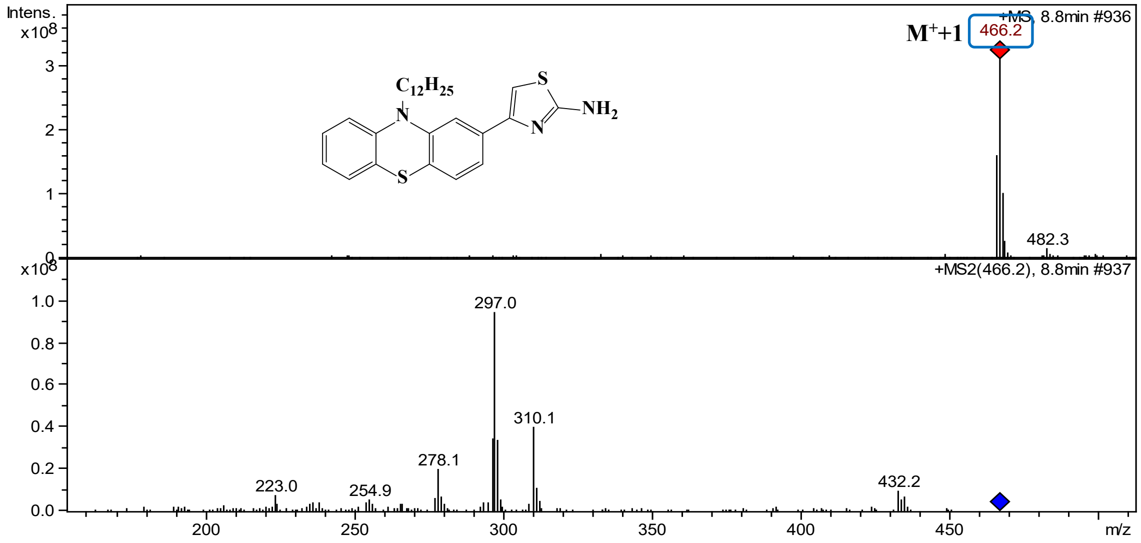

Figure S26. Mass spectrum of **A3.**

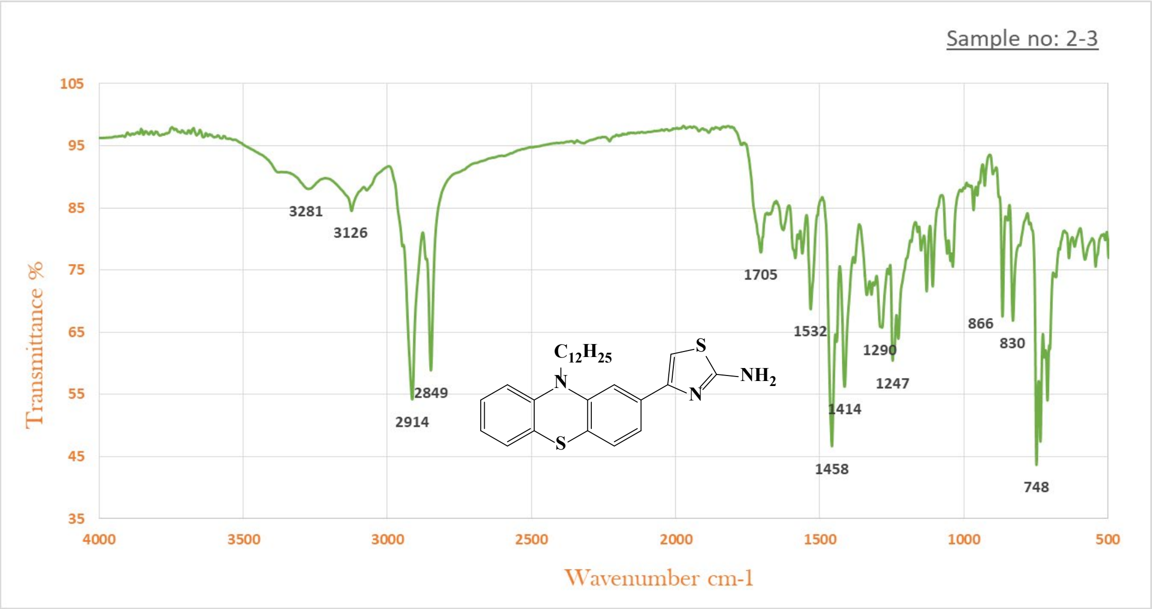

Figure S27. IR spectrum of **A3.**

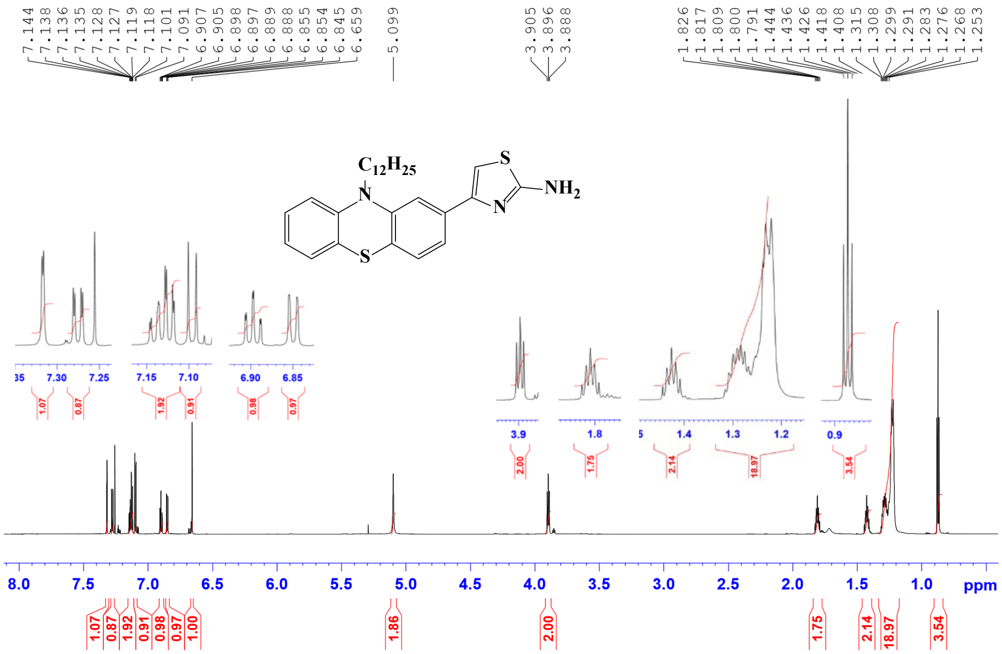

Figure S28. ^{1}H NMR ($CDCl_3$) spectrum of **A3.**

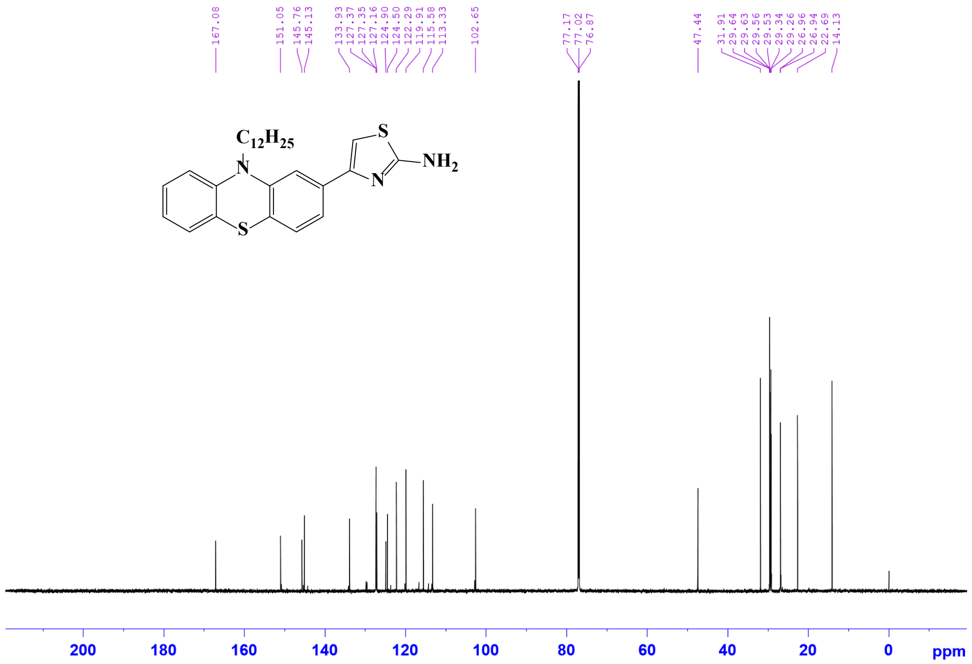

Figure S29. ^{13}C NMR ($CDCl_3$) spectrum of **A3.**

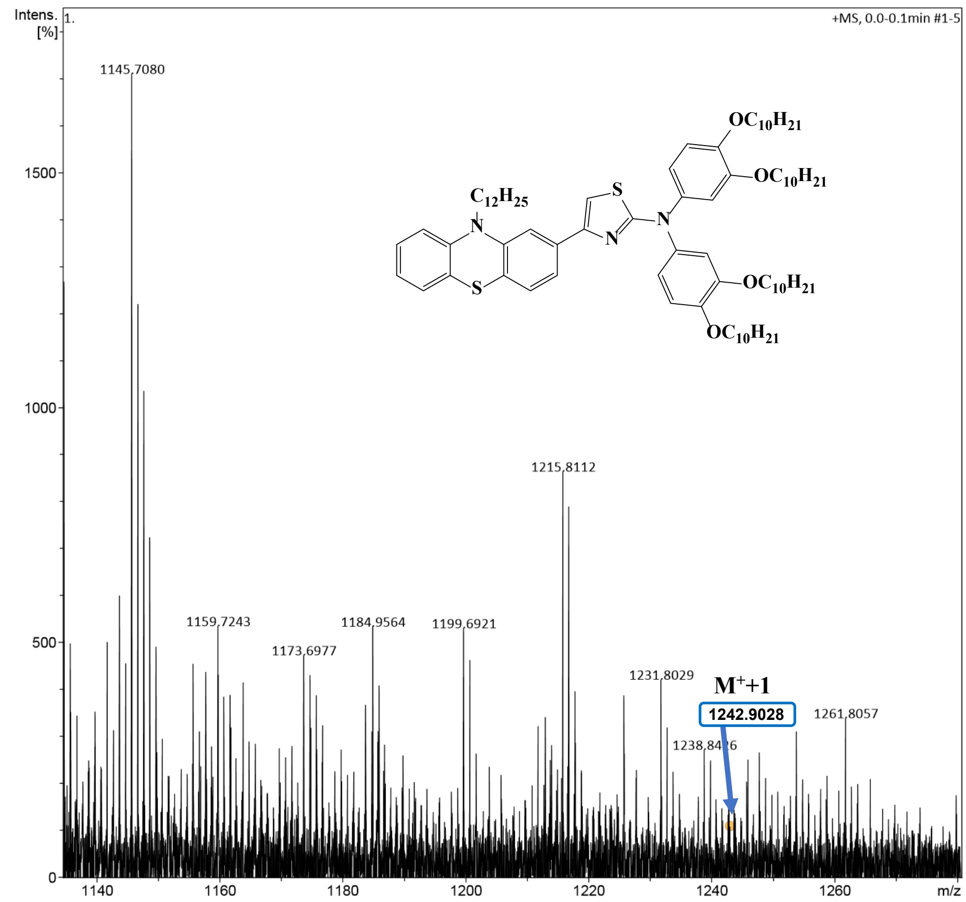

Cmpd 1, AutoMSn(301.1213), 0.1 min

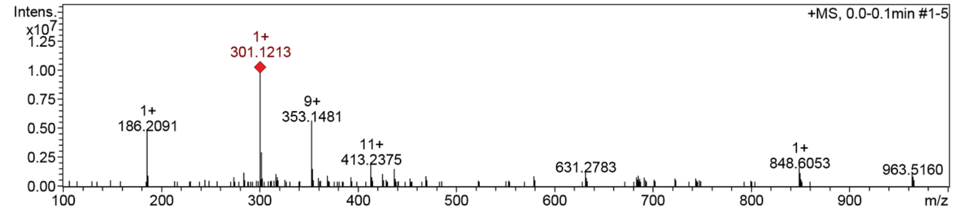

Meas. m/z	#	Ion Formula	m/z	err [ppm]	Mean err [ppm]	rdb	N-Rule	e‾ Conf	mSigma	Std I	Std Mean m/z	Std I VarNorm	Std m/z Diff	Std Comb Dev
1243.011397	1	C79H124N3O4S2	1242.902778	-87.4	-96.9	19.5	ok	even	377.9	298.5	n.a.	n.a.	n.a.	n.a.

Figure S30. High resolution mass spectrum of **compound 3.**

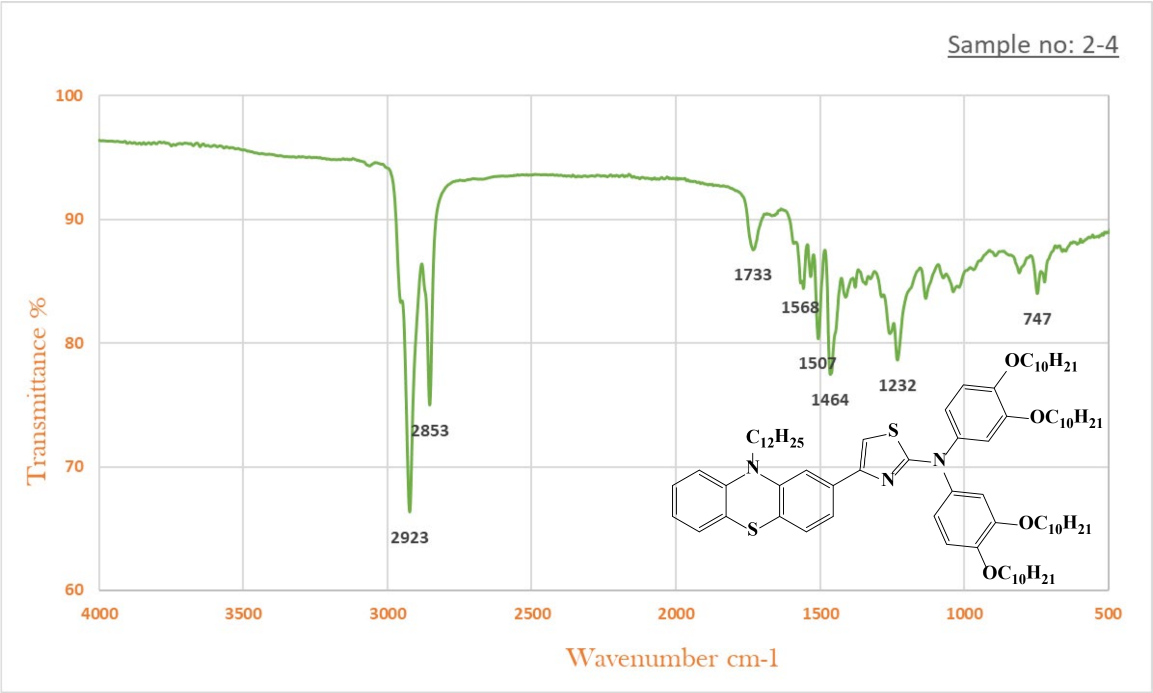

Figure S31. IR spectrum of **compound 3.**

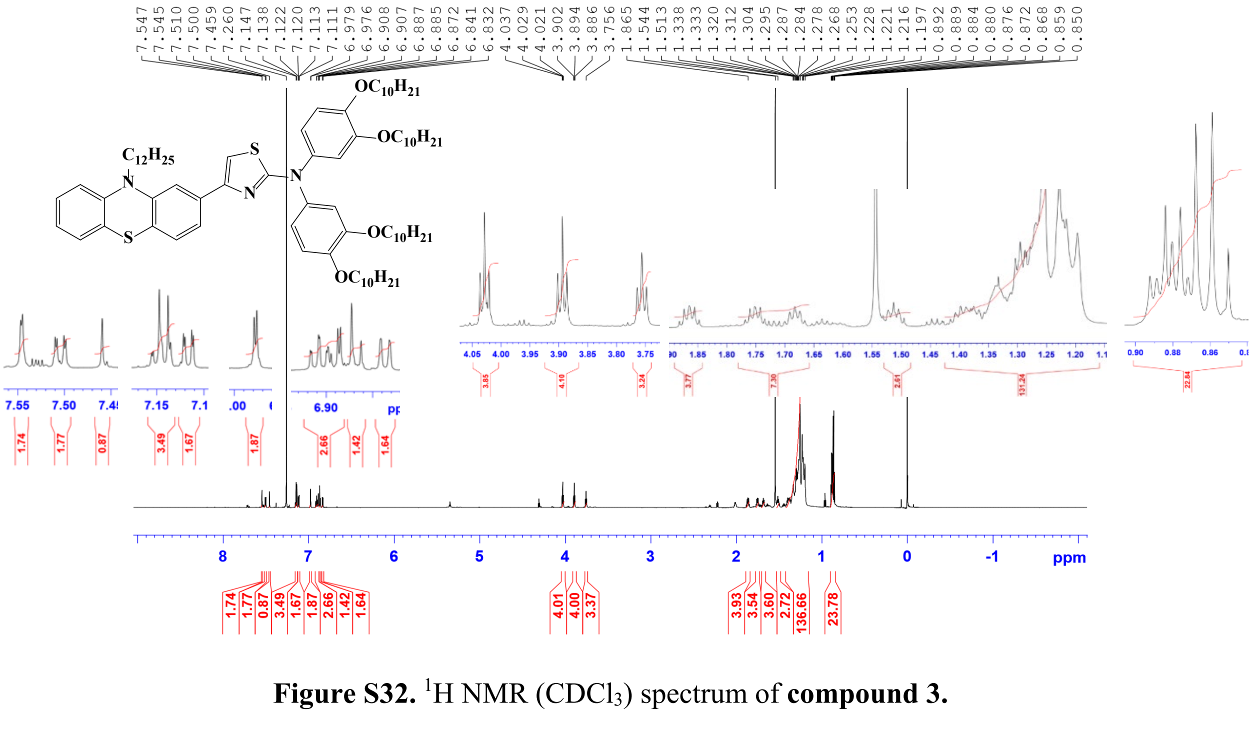

Figure S32. ^{1}H NMR ($CDCl_3$) spectrum of **compound 3.**

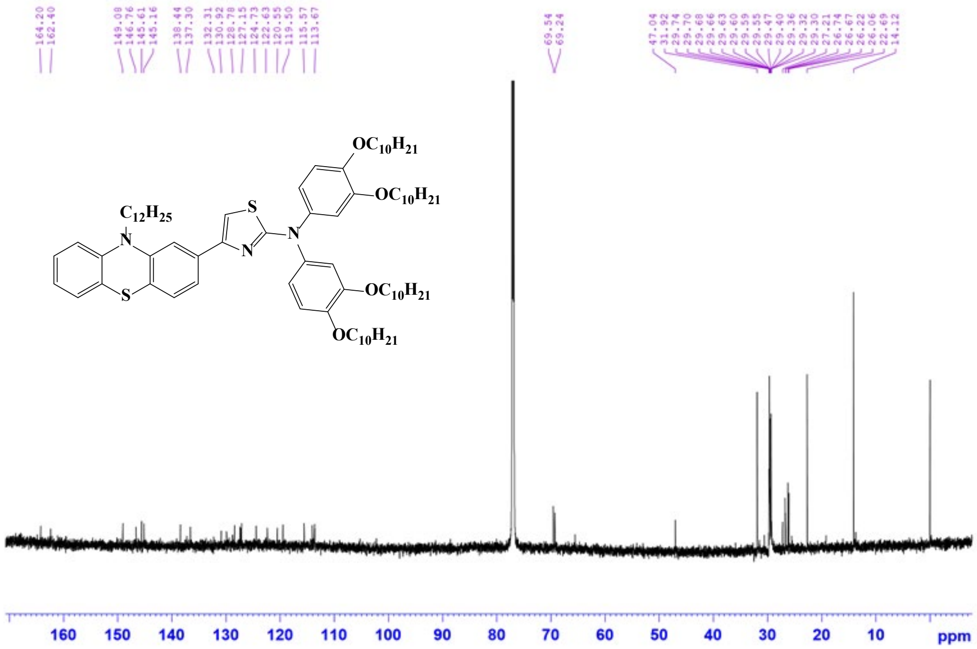

Figure S33. ^{13}C NMR ($CDCl_3$) spectrum of **compound 3.**

Table S1. UV-Vis Absorption properties of **Compounds 1, 2, and 3.**

Molecules	λ_1	λ_2	λ_3
**Compound 1**	229	300	-
**Compound 2**	229	299	-
**Compound 3**	229	256	292 (Shoulder)

Table S2. Bond lengths and dihedral angles of three compounds

	Compound 1	Compound 2	Compound 3
**Bond lengths (Å)**			
**L_1-L_2**	1.4246	1.4231	1.4360
**L_1-L_3**	1.4215	1.4201	1.3796
**L_1-L_4**	1.4201	1.4234	1.4329
**L_5-L_6**	/	/	1.4767
**Dihedral angles (°)**			
**ϕ_1**	41.6702	42.1668	16.3468
**ϕ_2**	39.8371	45.1419	46.0301
**ϕ_3**	46.2855	44.2488	60.1911
**ϕ_4**	/	/	6.9894

Table S3. Contribution with Partial density of states analysis.

Molecules	Orbitals	A	B	C	N	D
**Compounds 1**	HOMO	29%	22%	22%	26%	-
	LUMO	33%	25%	41%	0%	-
**Compounds 2**	HOMO	28%	27%	21%	25%	-
	LUMO	15%	29%	55%	1%	-
**Compounds 3**	HOMO	4%	6%	24%	7%	60%
	LUMO	3%	2%	44%	1%	50%

Table S4. The Vertical excitation energy (cm^{-1}), Absorption peak (nm), Oscillator strengths (*f*), Electron transitions and excited-state lifetime τ_2 (ns) of three investigated molecules.

Molecules	State	Energy	λ	*f*	CI	τ_2
**Compound 1**	S1	36013.97342	277.67	0.2767	H-L/0.66564	4.17686
**Compound 2**	S1	35953.11714	278.14	0.3412	H-L/0.64282	3.39875
**Compound 3**	S1	33034.91791	302.71	0.1619	H-L/0.57662	8.48415
